# Starfish Apaf-1 activates effector caspase-3/9 upon apoptosis of aged eggs

**DOI:** 10.1038/s41598-018-19845-6

**Published:** 2018-01-25

**Authors:** Ritsuko Tamura, Mariko Takada, Miki Sakaue, Ayaka Yoshida, Shirabe Ohi, Kaoru Hirano, Tomoyo Hayakawa, Noritaka Hirohashi, Kei Yura, Kazuyoshi Chiba

**Affiliations:** Department of Biology, Ochanomizu University, 2–1–1 Ohtsuka, Bunkyo-ku, Tokyo, 112–8610 Japan

## Abstract

Caspase-3-related DEVDase activity is initiated upon apoptosis in unfertilized starfish eggs. In this study, we cloned a starfish procaspase-3 corresponding to mammalian effector caspase containing a CARD that is similar to the amino terminal CARD of mammalian capsase-9, and we named it procaspase-3/9. Recombinant procaspase-3/9 expressed at 15 °C was cleaved to form active caspase-3/9 which has DEVDase activity. Microinjection of the active caspase-3/9 into starfish oocytes/eggs induced apoptosis. An antibody against the recombinant protein recognized endogenous procaspase-3/9 in starfish oocytes, which was cleaved upon apoptosis in aged unfertilized eggs. These results indicate that caspase-3/9 is an effector caspase in starfish. To verify the mechanism of caspase-3/9 activation, we cloned starfish Apaf-1 containing a CARD, a NOD, and 11 WD40 repeat regions, and we named it sfApaf-1. Recombinant sfApaf-1 CARD interacts with recombinant caspase-3/9 CARD and with endogenous procaspase-3/9 in cell-free preparations made from starfish oocytes, causing the formation of active caspase-3/9. When the cell-free preparation without mitochondria was incubated with inactive recombinant procaspase-3/9 expressed at 37 °C, DEVDase activity increased and apoptosome-like complexes were formed in the high molecular weight fractions containing both sfApaf-1 and cleaved caspase-3/9. These results suggest that sfApaf-1 activation is not dependent on cytochrome *c*.

## Introduction

Apoptosis plays important roles in metazoan development and tissue homeostasis^1–3^. It is executed by the activation of a family of aspartate specific cysteine proteases known as caspases^4^. Generally, inactive zymogens of caspases, known as procaspases, are synthesized constitutively and activated by specific proteolytic cleavage^4,5^.

In mammals, apoptosis pathways can be classified as either intrinsic or extrinsic^6^. The intrinsic apoptosis pathway is initiated by cellular stress, which releases cytochrome *c* from mitochondria into the cytoplasm^7,8^. Cytochrome *c* binds to the WD40 repeat regions of cytosolic apoptotic-protease-activating factor 1 (Apaf-1)^9^ to form a large complex known as the apoptosome^10,11^. Caspase-9, an initiator caspase, is recruited and activated by the apoptosome, and subsequently cleaves either procaspase-3 or -7 to make active effector/executioner caspase-3 or -7, respectively^12,13^. Association of procaspase-9 with Apaf-1 is mediated by their caspase recruitment domain (CARD) sequences located at the amino terminal^12,14^. The extrinsic apoptosis pathway is triggered by extracellular cell death stimulation such as death ligands^15^. Death receptors form the death-inducing signaling complex (DISC), and recruit initiator caspases, either caspase-8 or -10, followed by the activation of effector caspases^15–17^.

During apoptosis in the nematode *Caenorhabditis elegans*, the caspase-3 homologue, CED-3, is activated by the *C*. *elegans* apoptosome^18^. The apoptosome is formed by the Apaf-1 homolog, CED-4^19,20^, when CED-4 is released from the anti-apoptotic CED-9 by EGL-1^21,22^. CED-4 lacks WD40 repeat regions and does not require cytochrome *c* for apoptosome formation.

The *Drosophila melanogaster* caspase-9 homolog Dronc is activated by Dark, *D*. *melanogaster* Apaf-1 homolog^23,24^, which forms the fly apoptosome in the presence of dATP^25^. Cleaved Dronc subsequently activates the *D*. *melanogaster* caspase-3 homolog, Drice. Cytochrome *c* is not required for apoptosis in *D*. *melanogaster*, although Dark possesses WD40 repeat regions^26–28^.

DEVDase activity is sometimes used as a means to detect and identify effector caspases, whereas initiator caspases have activities independent of DEVDase. To the best of our knowledge, only five types of caspases were identified as effector caspases by using DEVDase of recombinant proteins, namely caspase-3 in vertebrates^29,30^, CED-3 in *C*. *elegans*^31^, Drice in *D*. *melanogaster*^32^, Cgcaspase-3 in Pacific oyster *Crassostrea gigas*^33^ and AmphiCASP-3/7 in amphioxus *Branchiostoma floridae*^34^, although direct activators of Cgcaspase-3 and AmphiCASP-3/7 have remained to be identified.

Meiosis reinitiation of oocytes in starfish (*Asterina pectinifera*) is stimulated by the hormone 1-methyladenine (1-MA), which is a prerequisite for fertilization. Without insemination or fertilization, endogenous caspase-3-like activity increases in aged eggs ∼10 h after 1-MA stimulation, followed by blebbing and apoptotic body formation^35–37^. Starfish eggs develop the competence to die when high extracellular signal-regulated kinase (ERK) activity is maintained for several hours^36,38^. After this ERK-dependent period, ERK is spontaneously inactivated, and apoptosis follows^36^. If starfish oocytes are not treated with 1-MA, they are alive over several days in seawater. Thus, hormonal stimulation leads to apoptosis, whereas fertilization blocks the apoptotic program.

As the starfish is one of the species located close to the evolutionary branching point between vertebrates and nematodes, it should help to provide clues to elucidate the relationship of apoptosis between vertebrates and nematodes. We therefore report here on the molecular mechanisms of starfish apoptosis, including the identification of caspase and its activation.

## Results

### Cloning and activity of starfish caspase-3/9 gene

**Figure 1:**
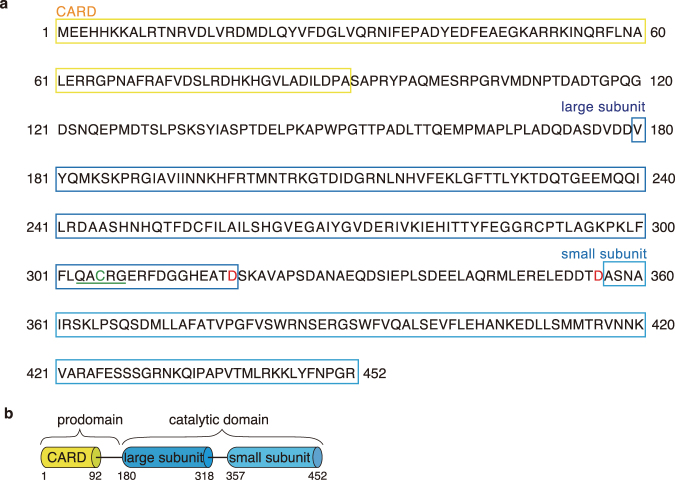
The sequence and the domain organization of caspase from starfish *A*. *pectinifera*. (**a**) Amino acid sequence of starfish caspase. The conserved active peptide region is underlined (green). The amino acids in red indicate the cleavage sites. (**b**) The domain organization of starfish caspase. The 1356 nt open reading frame (ORF) of starfish caspase encodes a protein of 452 aa, which exhibits a typical caspase-9 domain architecture containing amino terminal CARD (residues 1–92), large subunit (residues 180–318), and small subunit (residues 357–452).

**Figure 2:**
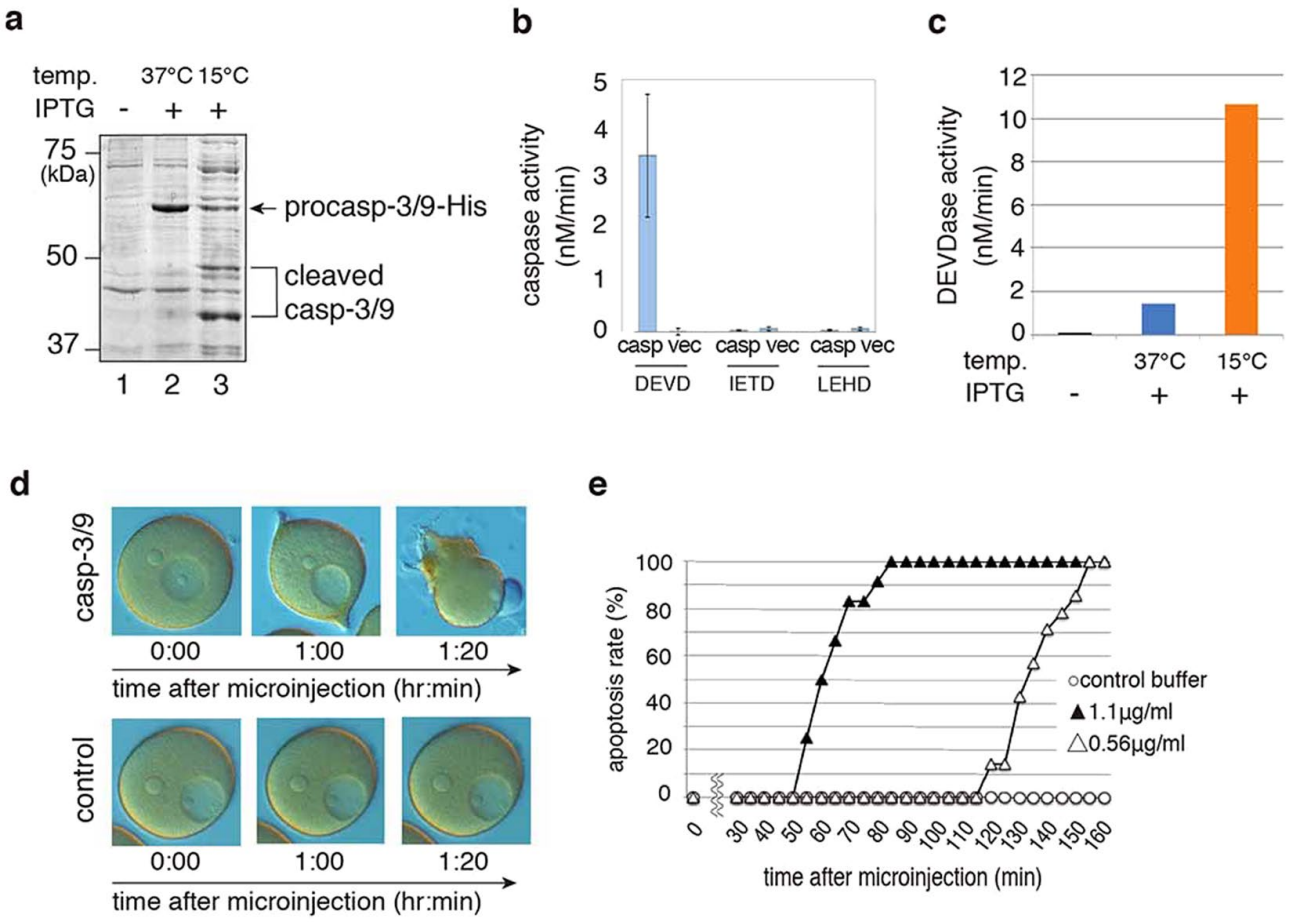
Expression of, and proteolytic activity assay for, recombinant caspase-3/9. (**a**) SDS-PAGE analysis of recombinant procaspase-3/9-His_6_ expressed in *E*. *coli* with CBB gel staining. Lanes: (1) No IPTG induction; (2) Procaspase-3/9 with IPTG induction at 37 °C; (3) Cleaved caspase-3/9 with IPTG induction at 15 °C. Full gel is presented in Supplementary Fig. S10. (**b**) Specific proteolytic activity of recombinant caspase-3/9-His_6_. Cell lysate from *E*. *coli* either transformed with a vector encoding caspase-3/9-His_6_ (casp) or control vector (vec) were analyzed for caspase-3 (DEVD), -8 (IETD), and -9 (LEHD) catalytic activity using Ac-DEVD-MCA, Ac-IETD-MCA and Ac-LEHD-MCA, respectively. (**c**) DEVDase activity of recombinant caspase-3/9-His_6_ expressed at different temperatures. Cell lysate from *E*. *coli* without IPTG induction, with IPTG induction at 37 °C, and with IPTG induction at 15 °C were analyzed for DEVDase activity using Ac-DEVD-MCA. (**d**) Microinjection of caspase-3/9-His_6_ into oocytes. Purified caspase-3/9-His_6_ (1.1 *µ*g/mL at a final concentration) or control buffer was microinjected into immature oocytes, and photographs were taken at the indicated times after microinjection. (**e**) The number of apoptotic eggs was counted after microinjection of purified caspase-3/9-His_6_ (closed triangle, 1.1 *µ*g/mL; open triangle, 0.56 *µ*g/mL at a final concentration) or control buffer (circle). The results are representative of four independent experiments.

In our previous studies, we detected caspase-3 (DEVDase) activity in unfertilized starfish eggs ∼30 min before blebbing^35,36,39^. To identify the responsible enzyme, we first cloned a caspase cDNA from a cDNA library of starfish ovaries using degenerate primers against caspase-3. We obtained a complete cDNA encoding a protein of 452 amino acids with a predicted molecular weight of 50.8 kDa. The deduced amino acid sequence contained the catalytic cysteine site (C305) in the pentameric QAC(R/G)G motif, which are perfectly conserved across species (Fig. 1a)^40^. Two putative cleavage sites are located at Asp318 and Asp356 (Fig. 1a). Comparing the predicted protein with those from other species using a BLAST search tool, we found that it exhibits a high level of sequence identity to effector caspases such as caspase-3 and -7, although it has a caspase recruitment domain (CARD) that is similar to those of initiator caspases such as caspase-2 and -9 (Fig. 1b). Starfish caspase-3/9 has caspase-9-like sequence in the N-terminal side, and caspase-3-like sequence in the C-terminal side (Supplementary Fig. S1 a and b). We hypothesized that the starfish caspase has properties of both initiator and effector caspase. To examine this possibility, we prepared recombinant starfish procaspase-His_6_. Full-length starfish procaspase (about 60.8 kDa in size) was observed at 37 °C (Fig. 2a, lane 2), whereas two cleaved fragments (about 40 kDa and 48 kDa) were detected at 15 °C (Fig. 2a, lane 3), suggesting that active recombinant caspase was formed at this temperature. The molecular weights of full-length caspase were slightly larger than the predicted procaspase (50.8 kDa), probably due to the presence of acidic amino acids^41^. When we mixed the cleaved caspase expressed at 15 °C with various substrates, it hydrolized only DEVD sequence that is recognized by an active mammalian caspase-3 (Fig. 2b), indicating that the activity of starfish caspase is very specific. Because starfish caspase has a caspase-9-like CARD and caspase-3-like DEVDase activity, we named it “caspase-3/9”. Recombinant procaspase-3/9-His_6_ expressed at 37 °C was rather inactive (Fig. 2c), suggesting that much proportion of the proteins has undergone unfolding at this temperature, as normal environmental temperatures for starfish is around 20 °C in seawater.

### Microinjection of recombinant caspase-3/9 into immature oocytes induces blebbing

To determine whether caspase-3/9 is sufficient to trigger apoptosis in starfish oocytes, we microinjected purified active caspase-3/9-His_6_ (1.1 or 0.56 *µ*g/mL final concentration) expressed at 15 °C or control buffer into the cytoplasm of immature oocytes. Blebbing was initiated within 1–2 h after microinjection of purified active caspase-3/9-His_6_ (Fig. 2d upper panels and 2e), whereas all oocytes injected with control buffer were alive (Fig. 2d lower panels and 2e). These results suggest that caspase-3/9 is an executor of apoptosis.

### Caspase-3/9 is cleaved and activated during apoptosis in unfertilized eggs

**Figure 3:**
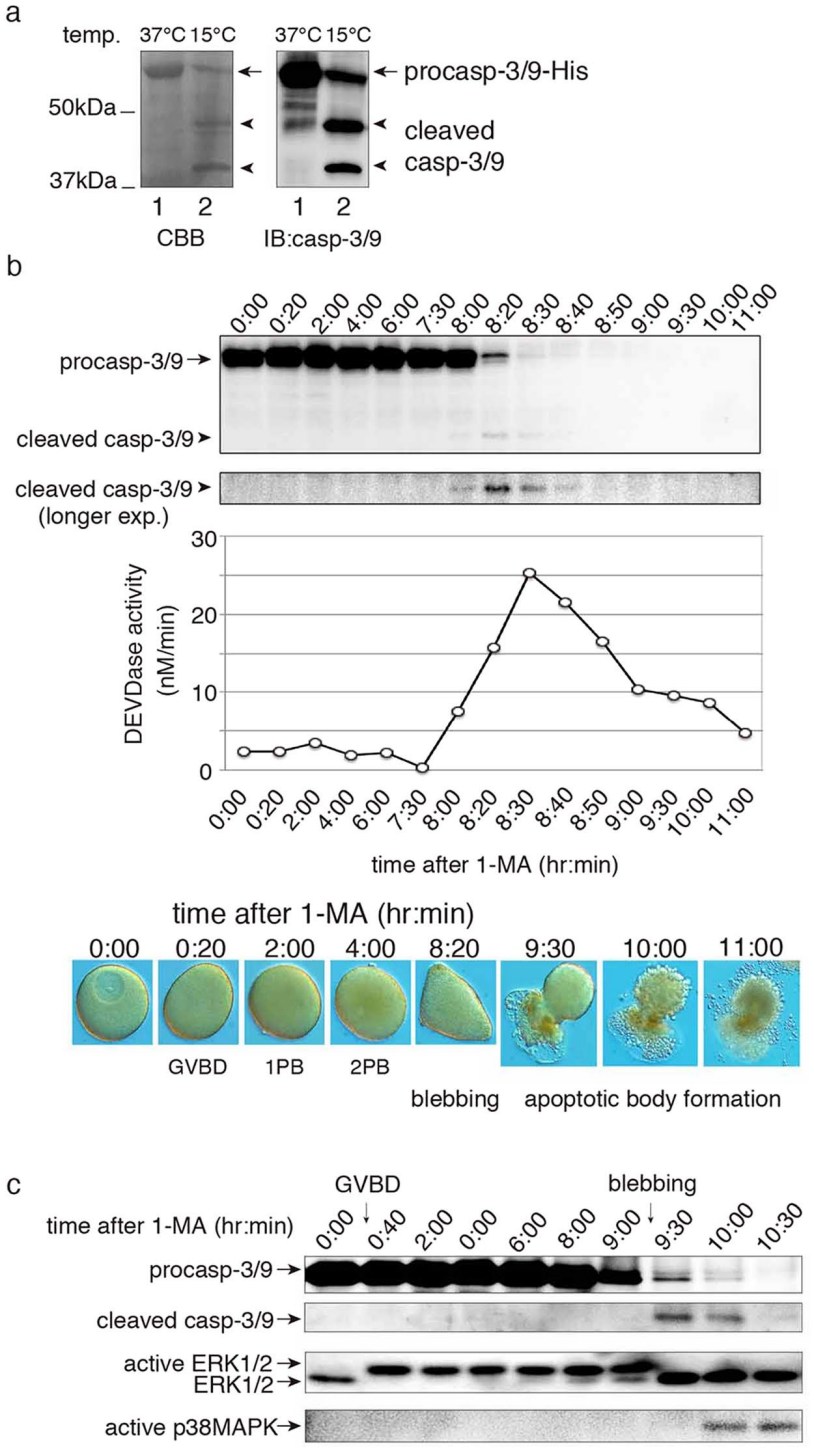
Activation and cleavage of endogenous caspase-3/9 upon apoptosis in unfertilized eggs. (**a**) CBB gel staining and western blotting analysis of recombinant caspase-3/9-His_6_. Cell lysate of *E*. *coli* expressing recombinant caspase-3/9-His_6_ was subjected to SDS-PAGE, followed by CBB gel staining (left panel), or analyzed by western blotting using the anti-caspase-3/9 antibody (right panel). Lanes: (1) with induction of IPTG at 37 °C; (2) at 15 °C. (**b**) Time course of endogenous caspase-3/9 activation after 1-MA treatment. Samples of oocytes were analyzed by SDS-PAGE and western blotting with the anti- caspase-3/9 antibody. Cleaved caspase-3/9 was visible after longer exposures. At the same time, the activity of endogenous caspase-3/9 was measured by the cleavage of Ac-DEVD-MCA. The morphological changes of the oocytes/eggs were observed with a light microscope equipped with Nomarski differential interference contrast optics; (0:00) immature oocyte; (0:20–4:00) mature eggs; (8:20) blebbing egg; (9:30–11:00) fragmented eggs. (**c**) Dynamics of caspase-3/9, ERK1/2 and p38MAPK during apoptosis. Samples were analyzed by western blotting with anti-caspase-3/9, anti-ERK1/2, and active p38MAPK-specific antibodies. Full gel and blots are presented in Supplementary Fig. S10. The results are representative of three independent experiments.

To verify that endogenous caspase-3/9 is involved in apoptosis in unfertilized starfish eggs, we first raised a specific antibody against recombinant procaspase-3/9-His_6_ (Fig. 3a). Using this antibody, we detected procaspase-3/9 by western blotting of oocytes/eggs before apoptosis (Fig. 3b, before 7:30). When we checked blebbing eggs with high DEVDase activity (Fig. 3b, after 8:00), we found that procaspase-3/9 was cleaved to form caspase-3/9. These results support the hypothesis that apoptosis in unfertilized eggs is caused by caspase-3/9.

In our previous studies, we reported that starfish apoptosis is induced by spontaneous inactivation of extracellular signal-regulated kinase (ERK) followed by activation of p38 MAPK^36^. Because artificial inactivation of ERK accelerated the timing of apoptosis^36^, we treated pre-apoptotic eggs with the MEK inhibitor U0126. As expected, apoptosis induction and procaspase-3/9 cleavage were observed earlier in the U0126-treated eggs than in the untreated eggs (Supplementary Fig. S2a and b). When we checked the timing of caspase-3/9 cleavage as well as inactivation/activation of ERK and p38 MAPK, we found that cleaved caspase-3/9 appeared after ERK inactivation, prior to p38 MAPK activation (Fig. 3c). Thus, it is likely that ERK inactivation induces the activation of both caspase-3/9 and p38 MAPK.

### Cloning of starfish Apaf-1

In mammalian apoptosis, the CARD of procaspase-9 interacts with the CARD of Apaf-1, which is followed by procaspase-9 cleavage and activation^13,14^. This caspase activation mechanism, including the formation of caspase multimers with Apaf- 1/CED-4/Dark, is conserved from nematodes to mammals^19^. As starfish caspase-3/9 has CARD, starfish eggs may express starfish Apaf-1, which would interact with caspase-3/9 CARD upon apoptosis.

**Figure 4:**
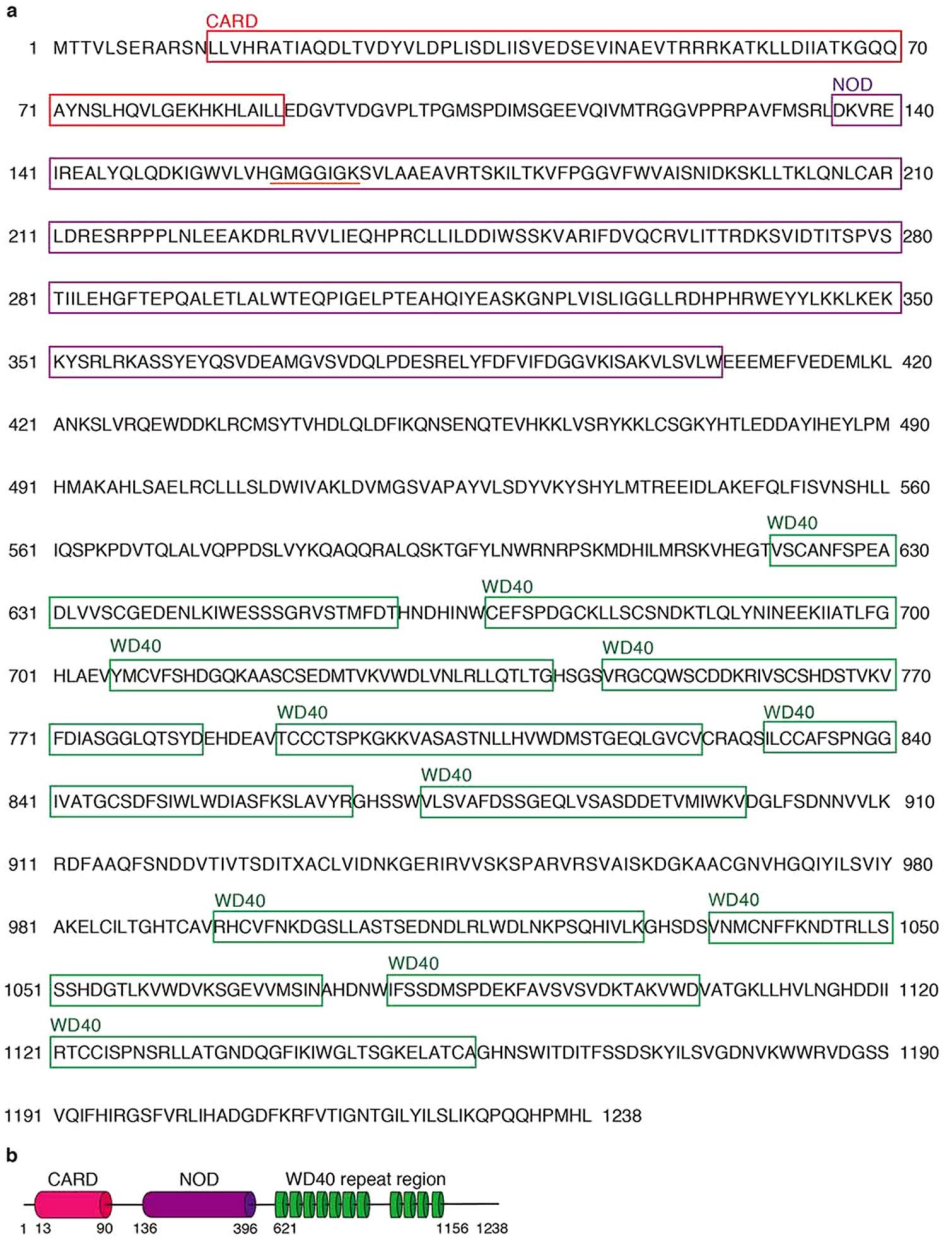
The amino acid sequence and the domain organization of sfApaf-1. (**a**) Amino acid sequence of sfApaf-1. The one putative nucleotide-binding site (GXXGXGK) is underlined (orange). sfApaf-1 contains three domains: CARD (red), NOD (purple), and 11 WD40 repeats (green). (**b**) Domain organization of sfApaf-1. The 3714 nt open reading frame (ORF) of starfish Apaf-1 encodes a 1238-aa protein with an amino terminal CARD (residues 13–90), NOD (residues 136–396), and WD40 repeat region (residues 621–1156).

To generate starfish *Apaf-1* cDNA, we used RT-PCR. The resulting complete cDNA encoded a protein of 1,238 amino acids with a predicted molecular weight of 138.5 kDa (Fig. 4a). Comparing the cDNA with other species using a BLAST search tool, it showed 36% identity with human *Apaf-1*, 23% identity with *D*. *melanogaster dark*, and 22% identity with *C*. *elegans ced-4*. These results strongly support the idea that the cDNA we generated encodes starfish Apaf-1, which is evolutionarily conserved (Supplementary Figs S3 and S4). Starfish Apaf-1 has one putative nucleotide-binding site (GXXGXGK) and several related motifs, CARD, a nucleotide-binding oligomerization domain (NOD), and 11 WD40 repeat regions (Fig. 4b). We predicted that starfish Apaf-1 interacts with caspase-3/9, causing activation of caspase-3/9 in a way similar to mammalian caspase-9.

### Starfish Apaf-1 binds to caspase-3/9 via a CARD-CARD interaction

**Figure 5:**
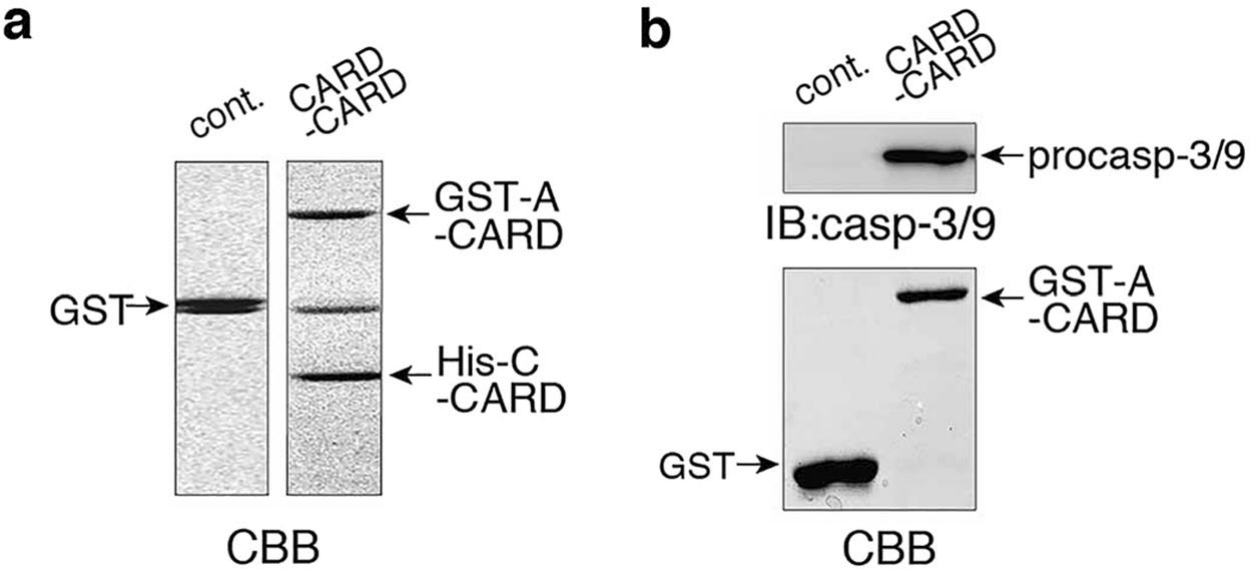
Interaction of sfApaf-1 CARD with caspase-3/9 CARD. (**a**) Glutathione Sepharose 4B bead-bound GST-A-CARD was incubated with His-C-CARD in PBS buffer; glutathione Sepharose 4B-bound GST was used as a control. Associated His-C-CARD was co-eluted with GST-A-CARD (right panel), but not with GST (left panel) after the addition of elution buffer, as determined by SDS–PAGE and CBB gel staining. (**b**) Glutathione magnetic agarose bead-bound GST-A-CARD or similarly bead-bound GST (control) was incubated with cell-free preparations containing endogenous procaspase-3/9. Endogenous procaspase-3/9 was pulled down with GST-A-CARD (right panel), but not with GST (left panel), and identified by western blotting with anti-caspase-3/9 antibody. Full blots are presented in Supplementary Fig. S10. The results are representative of two independent experiments.

To determine whether a CARD-CARD interaction occurs between starfish Apaf-1 (sfApaf-1) and caspase-3/9, we first expressed GST-sfApaf-1-CARD 1–134aa (GST-A-CARD) and His_6_-caspase-3/9-CARD 1–130aa (His-C-CARD). Either purified GST-A-CARD or control GST was bound to glutathione Sepharose 4B beads, followed by incubation with purified His-C-CARD. When the glutathione Sepharose 4B beads were subsequently treated with glutathione elution buffer, GST-A- CARD and His-C-CARD were co-eluted (Fig. 5a, right panel), whereas GST alone without His-C-CARD was eluted in the control experiment (Fig. 5a, left panel). These results indicate that A-CARD interacts with C-CARD. Next, we investigated whether recombinant A-CARD interacts with endogenous caspase-3/9 by pull-down assays using cell-free preparations made from starfish oocytes. GST-A-CARD and control GST beads were treated with cell-free preparation, and precipitated proteins with beads were analyzed by SDS–PAGE and western blotting with anti-caspase-3/9 antibody. We found that endogenous procaspase-3/9 in cell-free preparations was efficiently precipitated by purified GST-A-CARD, but not by GST alone (Fig. 5b). These results indicate that caspase-3/9 can interact directly with sfApaf-1 through their CARDs.

### Starfish Apaf-1 CARD associates with endogenous caspase-3/9

**Figure 6:**
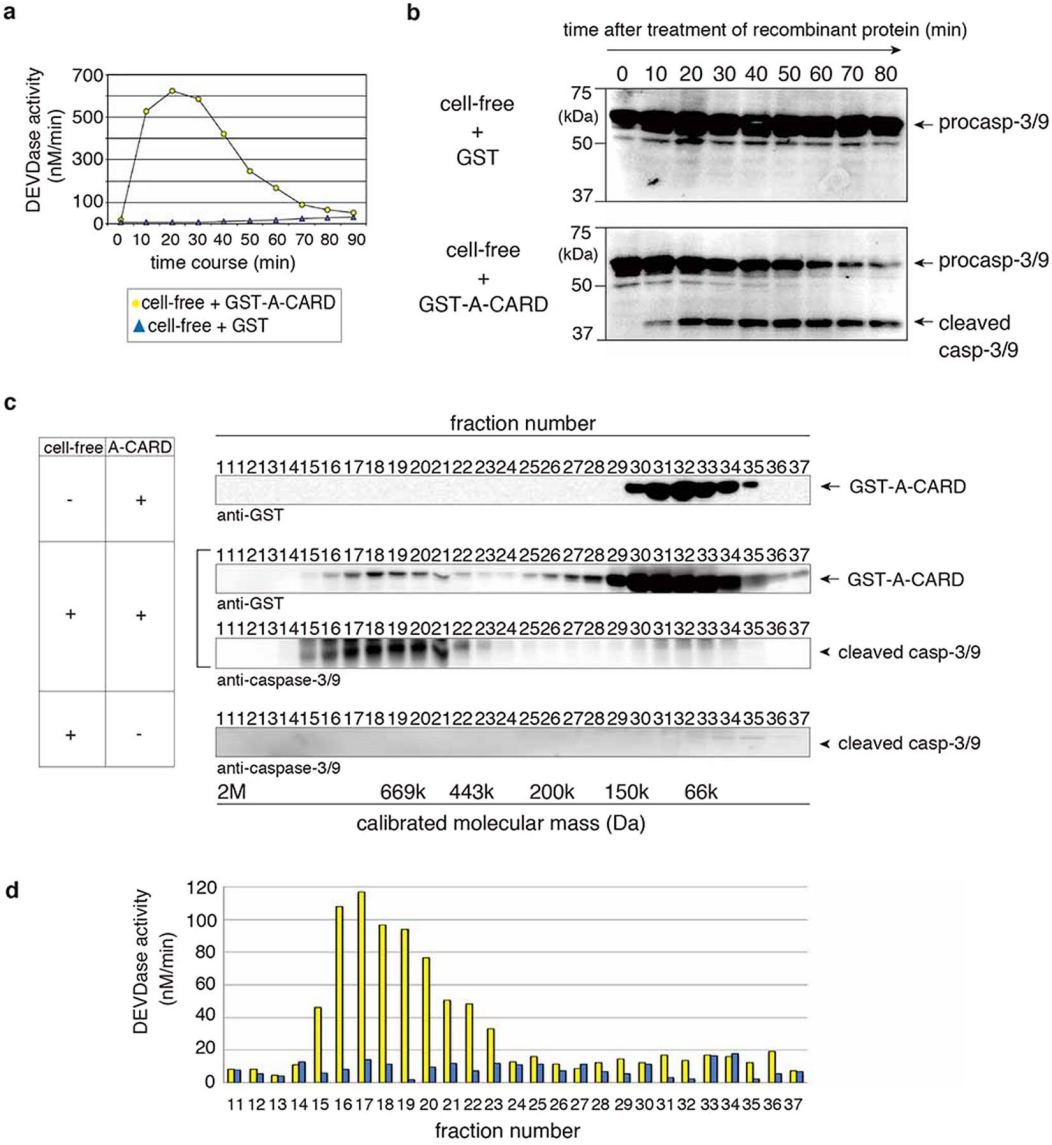
Activation of caspase-3/9 by sfApaf-1 CARD. (**a**) Activation of endogenous caspase-3/9 in cell-free preparation by GST-A-CARD. A time course of DEVDase activity was measured at the indicated time after addition of GST-A-CARD or control GST. (**b**) Cleavage of endogenous caspase-3/9 in cell-free preparations was induced by GST-A-CARD but not by GST, and detected by western blotting with the anti-caspase-3/9 antibody. (**c**) The interaction between A-CARD and caspase-3/9 in cell-free preparations. The cell-free preparation was incubated with GST-A-CARD or not, and fractionated by using gel filtration chromatography. Each fraction was analyzed by western blotting with anti-caspase-3/9 and anti-GST antibodies. (**d**) DEVDase activity in each fraction was measured by the cleavage of Ac-DEVD-MCA. The yellow column is from gel filtered cell-free preparations incubated with GST-A-CARD, and the blue column is from gel-filtered cell-free preparation without recombinant protein. Full blots are presented in Supplementary Fig. S10. The results are representative of three independent experiments.

Mammalian Apaf-1 associates with the apoptosome-activating caspase-9, and more importantly, the sole Apaf-1 CARD can increase caspase-9 activity by forming a large hetero-oligomer of Apaf-1-CARD/caspase-9 complex^42^. To examine whether GST-A-CARD can activate endogenous procaspase-3/9, we incubated purified GST-A-CARD in cell-free preparations made from starfish oocytes. We found that the DEVDase activity increased (Fig. 6a cell-free + GST-A-CARD) and endogenous procaspase-3/9 was cleaved in the presence of GST-A-CARD (Fig. 6b, bottom panel), but we detected no activity increase and no cleavage of procaspase-3/9 in the presence of the GST control (Fig. 6a cell-free + GST; 6b, upper panel). These results suggest that starfish Apaf-1 CARD activates caspase-3/9 in a manner similar to human Apaf-1 CARD. To examine whether GST-A-CARD in cell-free preparations made from starfish oocytes forms a large hetero-oligomer containing caspase-3/9, we performed the gel filtration analysis of GST-A-CARD incubated with or without the cell-free preparation. The GST-A-CARD (39.7 kDa) was eluted in a peak centered around the low molecular weight fraction 32 in the absence of the cell-free preparation (Fig. 6c, top), whereas it assembled into a large complex of roughly 0.7–1.4 MDa containing cleaved caspase-3/9 with high DEVDase activity (Fig. 6c: fractions 15–22 and 6d, yellow column). Instead, in the control gel filtration of cell-free preparation in the absence of A-CARD, the basal DEVDase activity of endogenous caspase-3/9 was low (Fig. 6d, blue column) without cleaved caspase-3/9 (Fig. 6c, bottom). Starfish Apaf-1-CARD therefore induces the formation of a large complex involving caspase-3/9, and the catalytic activity of caspase-3/9 is enhanced in this complex. In addition, these results suggest that endogenous procaspase-3/9 CARD is exposed in order to interact with exogenous sfApaf-1 CARD.

### sfApaf-1 is shifted from low to high molecular weight fractions by inactive procaspase-3/9-His_6_

**Figure 7:**
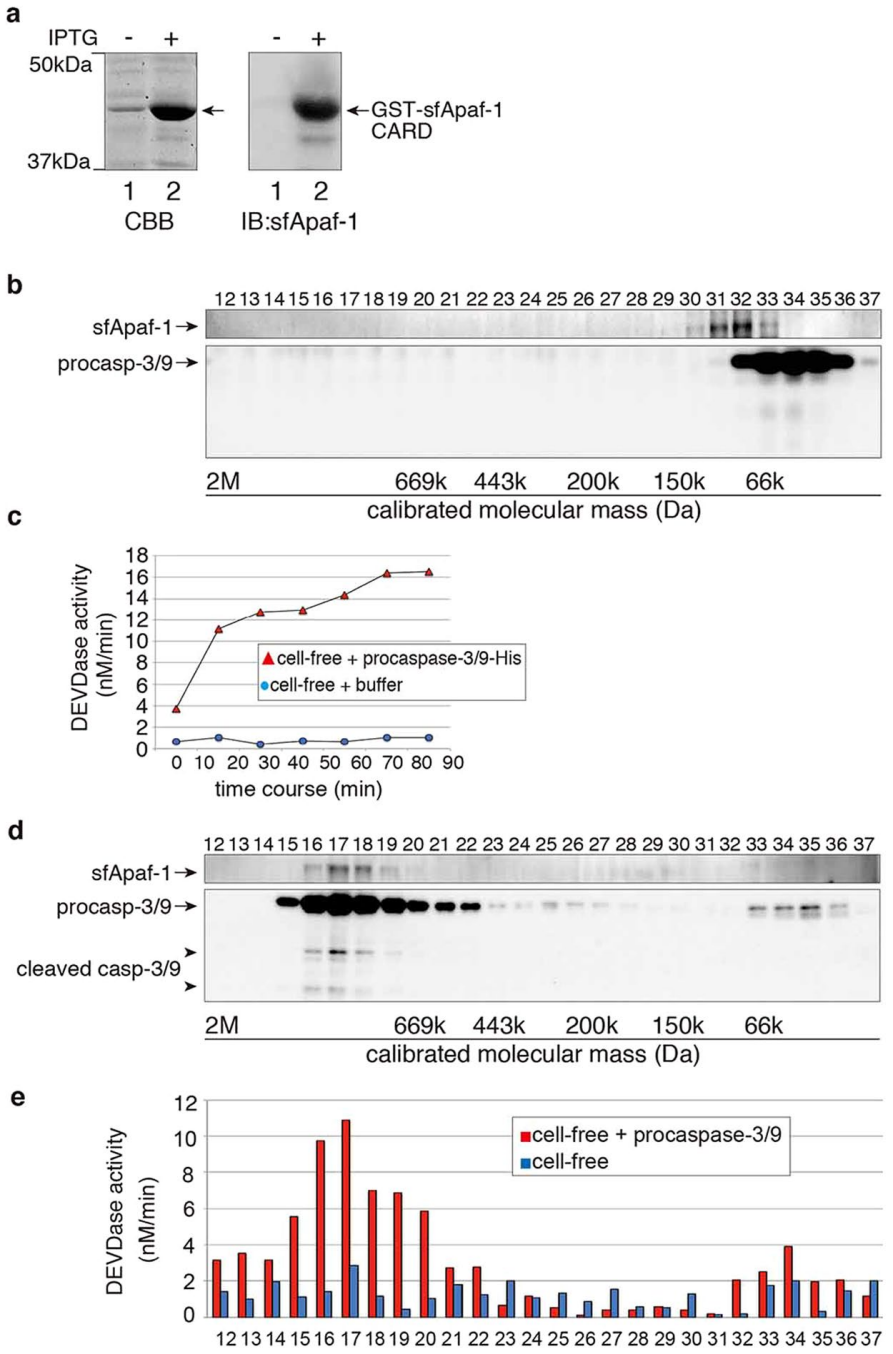
Apoptosome-like complex formation by recombinant procaspase-3/9-His_6_ in the cell-free preparations. (**a**) Western blot analysis of recombinant GST-sfApaf-1 CARD (1–134 aa) protein using anti-sfApaf-1 antibody. Lanes: (1) without IPTG induction; (2) with IPTG induction at 37 °C. (**b**) Ultracentrifuged cell-free preparations were fractionated by gel filtration chromatography. Endogenous procaspase-3/9 and sfApaf-1 were detected by western blotting with anti-caspase-3/9 and anti-sfApaf-1 antibodies. (**c**) Activation of endogenous caspase-3/9 in cell-free preparations by treatment with procaspase-3/9-His_6_. A time course of DEVDase activity was measured at the indicated times after adding either procaspase-3/9-His_6_ or buffer (control). (**d**) Ultracentrifuged cell-free preparations were incubated with recombinant procaspase-3/9-His_6_, and fractionated by gel filtration chromatography. Fractions were analyzed by western blotting with the anti-caspase-3/9 antibody and anti-sfApaf-1 antibody. (**e**) DEVDase activity in fractions was measured by the cleavage of Ac-DEVD-MCA. The red column is from gel filtered cell-free preparations with procaspase-3/9-His_6_, and the blue column is from gel filtered cell-free preparations without recombinant protein. Full blots are presented in Supplementary Fig. S10. The results are representative of two independent experiments.

To detect endogenous sfApaf-1, we raised a specific antibody against sfApaf-1. Because full-length sfApaf-1 was insoluble in *Escherichia coli*, we immunized rabbits with sfApaf-1 CARD (1–134aa) to obtain an anti-sfApaf-1 antibody (Fig. 7a).

To determine whether endogenous caspase-3/9 interacts with endogenous sfApaf-1 during apoptosis, we used a cell-free preparation^43^ to perform immunoprecipitation assays using anti-caspase-3/9 antibody. As expected, sfApaf-1 was efficiently co-immunoprecipitated with caspase-3/9 from the apoptotic cell-free preparation, but not from the non-apoptotic cell-free preparation (Supplementary Fig. S5). These results indicate that the interaction between caspase-3/9 and sfApaf-1 occurred upon apoptosis.

When the cell-free preparation without stimulation was gel filtered, followed by western blotting using the antibody against sfApaf-1 CARD, distribution of sfApaf-1 (Fig. 7b: fractions 31 and 32) was not always the same as that of procaspase-3/9 (Fig. 7b: fractions 32–36), suggesting that sfApaf-1 did not interact with procaspase-3/9 in the cell-free preparation before stimulation of apoptosis. Thus, it is likely that endogenous sfApaf-1 CARD was not exposed to interact with procaspase-3/9 CARD. Instead, endogenous procaspase-3/9 CARD should be exposed because GST-sfApaf-1-CARD could pull down procaspase-3/9 (Fig. 5b). In mammalian cells, Survivin-HBXIP (hepatitis B X-interacting protein) complexes or TUCAN bind procaspase-9, preventing procaspase-9 activation^44,45^. If procaspase-3/9 in starfish oocytes was blocked by such endogenous inhibitor proteins, recombinant procaspase-3/9-His_6_ may absorb the possible inhibitors suppressing activation of endogenous procaspase-3/9. As expected, DEVDase activity in the cell-free preparation increased after the addition of procaspase-3/9- His_6_ expressed at 37 °C (Fig. 7c), which was rather inactive initially (Fig. 2c). To determine whether dimerization of procaspase-3/9^46^ occurred in the cell-free preparation which had been incubated with inactive recombinant procaspase-3/9- His_6_, we performed gel filtration analysis. To our surprise, procaspase-3/9, cleaved caspase-3/9, and sfApaf-1 were eluted in the high molecular weight fractions corresponding to an apparent molecular weight of 0.7–1.4 MDa (Fig. 7d). Those fractions had DEVDase activity (Fig. 7e, red column), suggesting that sfApaf-1 formed the apoptosome. Because the apoptosome-like complex was formed in cell-free preparations, which had been ultracentrifuged to remove mitochondria, cytochrome *c* may not be required for sfApaf-1 activation. Correspondingly, endogenous procaspase-3/9 was not activated by cytochrome *c*/dATP addition to cell-free preparations (Supplementary Fig. S6), suggesting that starfish apoptosis is triggered by mechanisms other than cytochrome *c* release.

**Figure 8:**
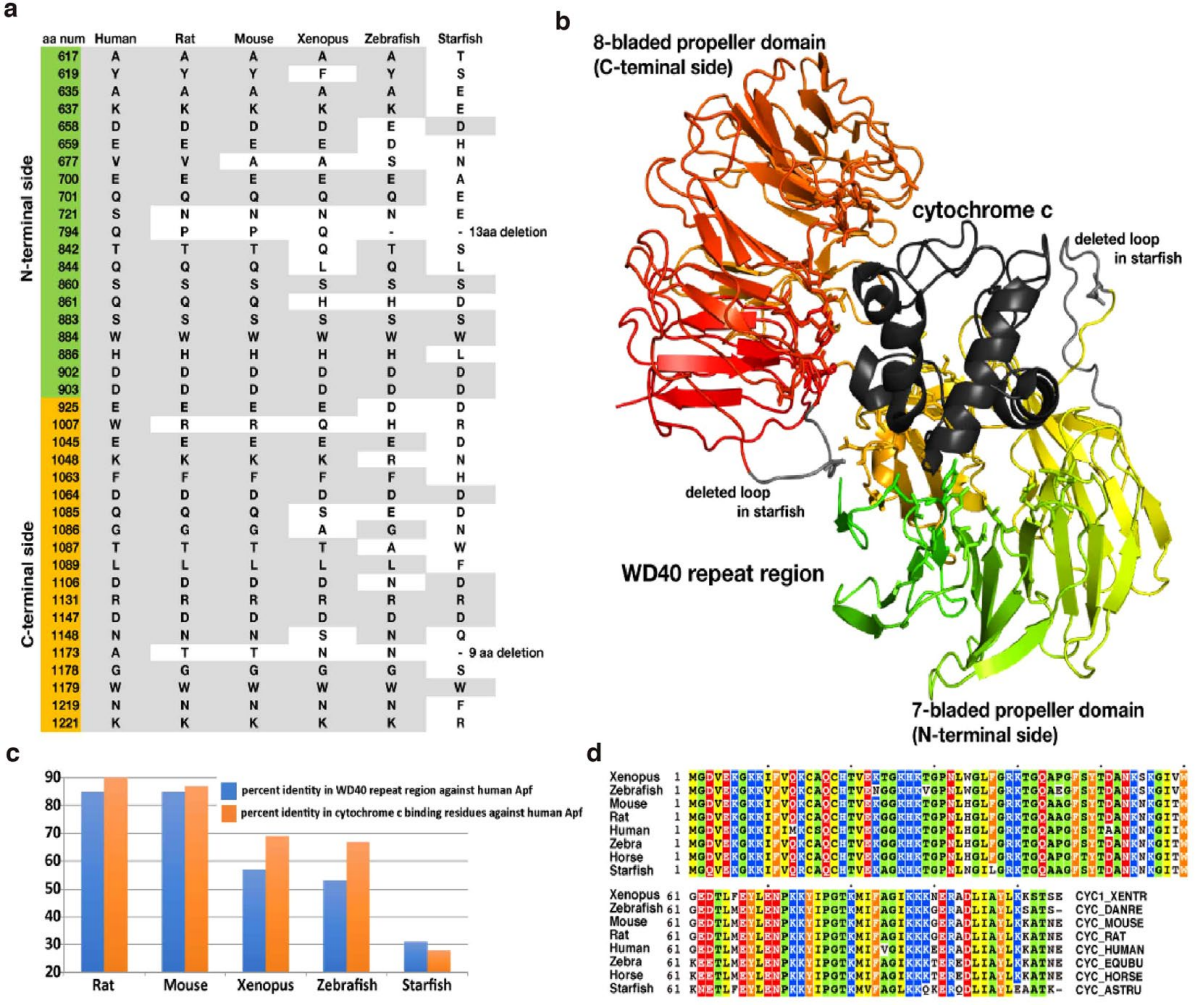
Structure of human Apaf-1 WD40 repeat in complex with horse cytochrome *c* and the characteristics of the interface. (**a**) Amino acid residues in WD40 repeats of vertebrate Apaf-1 that interact with horse cytochrome *c* and the corresponding residues in sfApaf-1. The residue in gray background has the same type of amino acid as in human Apaf-1. Note that sfApaf-1 has a long deletion in each propeller domain compared with Apaf-1 of other animals. (**b**) The three-dimensional structure of human Apaf-1 (WD40 repeat region) in complex with horse cytochrome *c* (PDB ID: 3jbt chains A and B). WD40 repeat region is colored from green to red and cytochrome *c* in black. Amino acid residues in WD40 repeat that interact with cytochrome *c* are depicted in stick model. The interaction Structure of human Apaf-1 WD40 repeat in complex with horse cytochrome *c* and the characteristics of the interface. The interaction was calculated based on ∆accessiblity and chose the residues that have difference in solvent accessible area, when the protein interacts with the partner or not. Two loops in gray protruding from WD40 repeat to cytochrome *c* are deleted in sfApaf-1. (**c**) Percentage identity in WD40 repeats (blue) and that in cytochrome *c* binding residues (orange) between Apaf-1 of human and other animals. Note that the values of percentage identity reverse in starfish Apaf-1. (**d**) The amino acid sequence alignment of cytochrome *c* from animals. The sequences were obtained from UniPort and the ID is shown at the end of each sequence. The sequence identities are between 73 (starfish and human) and 100 (rat and mouse) %, which are much higher than those in WD40 repeat of Apaf-1.

Zhou *et al*. (2015) determined the three-dimensional structure of human Apaf-1 in complex with horse cytochrome *c*^47^. The interactions were established between the WD40 repeat region of Apaf-1 and cytochrome *c* as a whole, and the specific amino acid residues involved in the interaction in the WD40 repeat region can be identified (Fig. 8a,b). The interface between the WD40 repeat region and cytochrome *c* mainly consists of hydrogen bonds and van der Waal’s contacts. The amino acid residues in the WD40 repeat region involved in the interface show high conservation in other vertebrates, but not in starfish (Fig. 8a). Thus, this low conservation of amino acid residues in the interface in the WD40 repeat region precludes a possibility of similar interactions, if any, between starfish Apaf-1 and cytochrome *c*. In addition, amino acid identities between the human and rat Apaf-1 interface interacting with cytochrome *c* were higher than that between the human and rat WD40 repeat region of Apaf-1 (Fig. 8c, left two columns). Similarly, in mice, frogs, and zebrafish, amino acid identities of the Apaf-1 interface interacting with cytochrome *c* were higher than those of the WD40 repeat region. However, in starfish, amino acid identities of the residues of sfApaf-1 corresponding to those in the human Apaf-1 interface were lower than that of the WD40 repeat region (Fig. 8c). These results indicate that conservation of the interface residues is high in vertebrates, whereas conservation of the surface residues of sfApaf-1 relating to the interface of Apaf-1 is low in starfish. This supports the hypothesis that sfApaf-1 does not interact with cytochrome *c*. The sequence identity of cytochrome *c* among different species including starfish is very high (Fig. 8d), which precludes the possibility of covariation between Apaf-1 and cytochrome *c* in starfish that could have evolved unique interactions between these proteins in starfish. Thus, the structural bioinformatics analysis has reached a conclusion that is consistent with the experimentally suggested scenario.

## Discussion

Stimulation of starfish oocytes by the hormone 1-MA is a prerequisite for fertilization and development. If mature eggs remain unfertilized, the 1-MA-mediated signaling pathway eventually triggers death via the activation of caspase-3-like DEVDase^36,39^. Although the 1-MA receptor has not been identified, it is likely to be a seven-transmembrane domain receptor without the mammalian Fas-like death domain, because 1-MA stimulation activates a heterotrimeric GTP-binding protein, which is sensitive to the pertussis toxin^48,49^. Starfish Gα i subsequently dissociates from Gβγ, which activates PI3-kinase^50,51^, and is followed by cdk1 and ERK activation^52^. Several hours after 1-MA stimulation, apoptosis is induced by spontaneous ERK inactivation^36,38^.

In this study, we identified the caspase having caspase-3-like DEVDase activity and named it caspase-3/9, because it encodes sequences approximately 29% identical to CARD of mammalian caspase-9. Interestingly, starfish caspase-3/9 (Deuterostome) was relatively close to CED-3; *C*. *elegans* effector caspase with a CARD (Protostomes)^31^. These results suggest the evolutionary scenario that an ancient caspase was the “CARD-effector” type having DEVDase activity (Supplementary Figs S7 and S8)^29–34,53–55^. The ancient CARD-effector caspases might have undergone subfunctionalization after gene duplication in vertebrates and arthropods, dividing the functions of effector and initiator caspases into two different caspases (Supplementary Fig. S8). Vertebrate caspase-3 has a role in amplifying the apoptotic signal from initiator caspase^46^, whereas starfish caspase-3/9 may have an auto-amplification ability, because active caspase-3/9 and sfApaf-1 were eluted in high molecular weight fractions (Fig. 7).

The procaspase-3/9 was cleaved upon apoptosis in unfertilized eggs, and injection of recombinant caspase-3/9 induced apoptosis in oocytes. Thus, caspase-3/9 has a role as an effector/executioner caspase. Because the CARD-CARD interaction was observed between recombinant CARDs of caspase-3/9 and sfApaf-1, caspase-3/9 has function akin to initiator as well as effector caspases (Figs 5,6). Similarly, *C*. *elegans* CED-3 is an effector caspase attached with CARD^18,31^.

Mammalian Apaf-1, Apaf-1 homologs of *C*. *elegans* CED-4, and *D*. *melanogaster* Dark interact with their procaspases through CARDs, and activate caspases^12,14,28,31^. The regulation of Apaf-1, however, differs significantly from the regulation of Apaf-1 homologs. In mammals, cytochrome *c*, which is released from mitochondria, binds to the WD40 repeat region at the carboxyl terminus of Apaf-1^7–10^. This binding is a trigger for forming the apoptosome and activating procaspase-9^13,14^. On the other hand, no cytochrome *c* is required for the regulation of either CED-4 or Dark^20,21,27,28^. Because DEVDase activity was not increased by the addition of cytochrome *c*/dATP to cell-free preparations made from starfish oocytes (Supplementary Fig. S6) and procaspase-3/9 was activated in ultracentrifuged cell-free preparations lacking mitochondria (Figs 6 and 7), sfApaf-1 apparently can be activated without cytochrome *c* during starfish egg apoptosis. The structural and bioinformatics analyses support this hypothesis (Fig. 8).

Before the CARD-CARD interaction, mammalian procaspase-9 and its *D*. *melanogaster* procaspase-9 homolog, pro-Dronc, are inactivated by the binding of inhibitors such as Survivin-HBXIP complexes or TUCAN, and DIAP1^44,45,56^. The release of such inhibitors is required for activating procaspases. When inactively expressed procaspase-3/9-His_6_ at 37 °C was incubated in cell-free preparations, DEVDase activity increased and cleavage of procaspase-3/9 occurred (Fig. 7). These results suggest that an inhibitor for procaspase-3/9 may be absorbed by the recombinant protein, causing the dimerization and activation of endogenous procaspase-3/9 as demonstrated for mammalian procaspase^46^.

In mammals, the ERK pathway is associated with the regulation of apoptosis^57^, and ERK2 inhibits caspase-9 by direct phosphorylation at Thr125^58,59^. In starfish, capsase-3/9 has a predicted ERK2 phosphorylation site (Thr153) (Supplementary Fig. S9), suggesting that procaspase-3/9 phosphorylation may regulate caspase-3/9 activation. Indeed, procaspase-3/9 was activated after ERK inactivation in unfertilized oocytes (Fig. 3c). In addition, we showed in this study that activation of caspase-3/9 in starfish unfertilized eggs was regulated by ERK, and membrane blebbing followed ∼2 h after spontaneous inactivation of ERK^35^. Further studies are needed to verify the mechanism of caspase-3/9 activation.

## Materials and Methods

### Animals and oocyte preparation

*Asterina pectinifera* were collected on the Pacific coast of Japan, and were kept in laboratory aquaria with filtered seawater at 14–15 °C. To remove follicle cells, oocytes released from isolated ovaries were washed three to five times with ice-cold Ca^2 + ^-free seawater (CFSW: 450 mM NaCl, 9 mM KCl, 48 mM MgSO_4_, 6 mM NaHCO_3_, 40 mM EPPS, pH 8.0), and incubated in artificial seawater (ASW: 450 mM NaCl, 9 mM KCl, 48 mM MgSO_4_, 6 mM NaHCO_3_, 40 mM EPPS, 9.2 mM CaCl_2_, pH 8.0) at 20 °C. Oocyte maturation was induced by treatment with 1uM 1-MA. After 100% germinal vesicle breakdown was verified, 1-MA was washed out. Eggs were incubated in ASW at 20 °C.

### cDNA cloning of procaspase-3/9

All ovaries collected from a starfish were homogenized, and poly(A) + RNA was made using FastTrack2.0 (Invitrogen). Both cDNA and cDNA library were made by using SuperScript Plasmid System and Plasmid Cloning with Gateway^TM^ Technology (Thermo Fisher Scientific) and the pSPORT 1 vector. The cDNA encoding capsase-3/9 was cloned from cDNA library by PCR screening using degenerate primers specific for conserved sequences in caspase-3 (5′-ATCATHAAYAAYAARAAYTTYSA-3′ and 5′-GCCTGRATRAARAANAGTTTRGGYTT-3′) and Taq DNApolymerase (TaKaRa).

### cDNA cloning of sfApaf-1

To clone sfApaf-1, total RNA from starfish ovaries was extracted using RNA Wiz (Amnion). First-strand cDNA was made by using random 9 primer (TaKaRa) and PrimeScriptII Reverse Transcriptase (TaKaRa). PCR was performed with degenerate primers for *Apaf-1* corresponding to the amino acid sequences of Apaf-1 orthologues from *H*.*sapiens* (116–123, 437–446), *Xenopus laevis* (116–123, 437–446), *Branchiostoma floridae* (115–122, 438–447), *Strongylocentrotus*

*purpuratus* (123–130, 447–456), *Echinacea pallida* (106–113, 426–435), and *D*. *melanogaster* (116–123, 439–448) (1st; 5′- GGNGGNGTNCCNATNCCICC-3′ and 5′-ARRAARTCNARYTGNARRTYRTG-3′, 2nd; 5′-CATGGNATGGGNGGNATNG

GIAAR-3′ and 5′-ARRTARTAYTTCCANCKIKTIGG-3′) using the *Ex Taq* system (TaKaRa). The 552 bp PCR product was sequenced after subcloning into the pCRII-TOPO vector (Invitrogen) using the TA cloning kit (Invitrogen). We subsequently designed specific primers based on the PCR products. A partial *sfApaf-1* was obtained by performing PCR with specific primers and degenerate primers. To isolate full-length *sfApaf-1*, the 5′ and 3′ end was identified by performing rapid amplification of cDNA ends (RACE) using the 5′-Full RACE Kit and 3′-Full RACE Core Set (TaKaRa). The specific primers for RACE were designed based on the partial *sfApaf-1* sequence obtained above. For 5′ RACE, total RNA was transcribed into cDNA with specific primers (5′-[Phos]CTCAATCTATCCTT-3′) using AMV Reverse Transcriptase (Promega), and used as a template. Following RNA degradation, first-strand cDNA was circularized using T4 RNA ligase. PCR was performed with specific primers (1st; 5′-CACAGGTTCTGTAGCTTGGT-3′ and 5′-AGACTAGACCGTGAGTCC-3′, 2nd; 5′-CTGATGGCGACCCAGAACAC-3′ and 5′-CCCACTCAACCTGGAGGAG-3′) using the *Ex Taq* system (TaKaRa). The

resulting PCR product was cloned into pCRII-TOPO vector (Invitrogen) using TOPO TAcloning kit (Invitrogen) and sequenced. For 3′ RACE, total RNA was transcribed into cDNA with 3′ RACE adaptor and used as a template. PCR was subsequently performed with specific primers (1st; 5′-ATAAGCTGCGCTGCATGA-3′, 2nd; 5′-CAGGAATGGGATGATGATAAGCT-3′) and an adaptor primer. The resulting PCR product was cloned into the pCRII-TOPO vector (Invitrogen) using TOPO TA Cloning Kit (Invitrogen) and sequenced. All primer sequences are shown in supplementary Table S1.

### Recombinant protein preparation

C-CARD (residues 1–130) was expressed in *Escherichia coli* strain BL21(DE3) (TaKaRa) as N-terminally His-6-tagged proteins by using the pET-23b(+) vector (Novagen). A-CARD (residues 1–130) and partial sfApaf-1 (residues 1–541) were expressed in *E*. *coli* BL21(DE3) bacteria as N-terminally GST-tagged proteins by using pGEX-6P-3 vector (GE Healthcare). Fresh 300 mL cultures were grown to OD_600_ = 0.4 at 37 °C, and incubated with 1 mM IPTG (Sigma) for 1.5 h. Bacterial pellets were lysed in 30 mL of lysis buffer (100 mM KCl, 20 mM HEPES-KOH, pH 7.5), and homogenized by sonication (Sonifier S-250A analog ultrasonic processor). It was centrifuged at 8000 g for 10 min, and the supernatant was stored at −80 °C. His-C-CARD was purified by TALON Metal Affinity Resin (TaKaRa), GST-A-CARD was purified using glutathione Sepharose 4B (GE Healthcare).

Recombinant, full-length caspase-3/9 was expressed in BL21(DE3) pLysS bacteria (TaKaRa) with a C-terminal His-6 tag by using pET-23b(+) vector (Novagen). Fresh 150 mL cultures were grown to OD_600_ = 0.4 at 37 °C. Inactive procaspase-3/9 was induced with 1 mM IPTG for 1.5 h at 37 °C. Active caspase-3/9 was induced with 1 mM IPTG for 12 h at 15 °C. Bacterial pellets were lysed in 30 mL lysis buffer, and homogenized by sonication. They were centrifuged at 15000 g for 30 min, and supernatant was stored at −80 °C. Recombinant caspase-3/9 was purified using TALON Metal Affinity Resin.

### Caspase proteolytic activity assay

Caspase activity was determined by the cleavage of the peptide substrates Ac-DEVD- MCA, Ac-IETD-MCA, and Ac-LEHD-MCA (Peptide Institute, Inc., Osaka, Japan). The substrates dissolved in dimethyl sulfoxide at 10 mM were added to the samples to a final concentration of 0.1 mM. Fluorescence intensity was measured at 380 nm for excitation and at 460 nm for emission using FluoroMax-4 (HORIBA, Ltd., Kyoto, Japan).

### SDS–PAGE and western blotting

Eggs were collected at various time points after 1-MA treatment (0–11 h). Eggs (n = 60) in 60 µL sample buffer^60^ were boiled for 5 min at 95 °C. Samples containing 10 eggs were subjected to 12.5% SDS-PAGE. Proteins were blotted onto PVDF membranes (Immobilon-P, 0.45 *µ*m, Millipore). Each membrane was blocked with PBS–T (0.05% Tween20-PBS) containing 1% BSA (Sigma-Aldrich), and was incubated with the anti-caspase-3/9 antibody, anti- ERK1/2 antibody (CST), and anti-active p38MAPK antibody (Promega) at a dilution of 1:2000 for 1 h at room temperature. After washing three times with PBS-T, membranes were incubated with the second anti-rabbit HRP antibody at a dilution of 1:2000 for 1 h at room temperature. After washing twice with PBS-T for 10 min and once with PBS for 10 min, proteins were detected using ECL Prime Western Blotting Detection System (GE Healthcare) and LAS-4000mini Luminescent image analyzer (Fuji Photo Film Co.). The results were analyzed by Image Gauge software (Fuji Photo FilmCo.).

### Microinjection

Microinjections into oocytes and quantitation of injection volumes were performed according to the methods of Hiramoto^61^. Oocytes were held between two coverslips separated by two pieces of double-sided tape during microinjection and observation^48^.

### Preparation of the oocyte homogenate and supernatant

Cell-free preparations were made as described previously^43^. De-jellied immature oocytes or mature eggs were washed twice in 10 volumes of ice-cold P11 buffer (150 mM Glycine, 100 mM EGTA, 200 mM HEPES-KOH, pH 7.0). After P11 buffer was removed, oocytes were homogenized by passing through a nylon mesh and centrifuged at 20000 g for 15 min at 4 °C. The supernatant was frozen with liquid nitrogen, and stored at −80 °C until use. For the pull-down assay and gel filtration analysis, cell-free preparation was 3-fold diluted with P11 buffer, and ultracentrifuged at 65000 g for 1 h. The supernatant was frozen with liquid nitrogen, and stored at −80 °C.

### Assay for CARD-CARD interaction

Purified GST-A-CARD (2 nmol) and GST (2 nmol) were incubated with 100 µL washed Glutathione Magnetic Agarose Beads (Thermo Fisher) for 30 min at room temperature, and washed three times with 300 µL wash buffer according to the protocol. GST-A-CARD or GST beads were mixed with purified recombinant His-C-CARD (4 nmol) in PBS buffer. They were incubated for 30 min at room temperature, and magnetic beads were washed twice with 300 µL wash buffer to remove unbinding proteins. Bound proteins were eluted with 100 µL elution buffer, and CARD-CARD interactions were detected by SDS-PAGE with CBB staining.

### Pull-down assay

Purified GST-A-CARD (2 nmol) and GST (2 nmol) were incubated with 100 µL washed Glutathione Magnetic Agarose Beads (Thermo Fisher) for 1 h at room temperature, and washed three times with 300 µL wash buffer. Each bead was mixed with ultracentrifuged cell-free preparations made from immature oocytes. They were incubated for 30 min at 4 °C, and precipitated magnetic beads were washed twice with 300 µL wash buffer. Sample buffer (100 µL) was added to the washed beads and boiled for 5 min at 95 °C, followed by SDS-PAGE and western blotting with the anti-caspase-3/9 antibody. Immunoprecipitation was performed as described ^62^.

### Gel filtration analysis

Purified GST-A-CARD at a 2 mM final concentration was incubated with 800 µL ultracentrifuged cell-free preparation from immature oocytes for 30 min at room temperature. Gel filtration was performed by using a Superose^TM^ 6 10/300 GL column (GE Healthcare) with the gel filtration buffer containing 10 mM HEPES (pH 7.5), 100 mM NaCl, and 2 mM dithiothreitol at 4 °C. The column was calibrated with molecular weight standards.

Purified procaspase-3/9-His_6_ at the final concentration of 3 mM was incubated with 800 µL ultracentrifuged cell-free preparation from immature oocytes for 30 min at room temperature. Gel filtration steps are same as the above.

### Accession codes

The data present in this work was deposited in NCBI’s Gene Expression Omnibus (GEO) database under the accession number ACM46824 (caspase-3/9) and MF612046 (sfApaf-1).

## Electronic supplementary material

Supplemental Figures, and Materials & Methods
