# Supplementary material for: Starfish Apaf-1 activates effector caspase-3/9 upon apoptosis of aged eggs
Source: Sci Rep. 2018 Jan 25;8:1611. doi: 10.1038/s41598-018-19845-6 (PMC5785508; doi:10.1038/s41598-018-19845-6)
Supplement: Supplementary file 1 — Supplemental Figures, and Materials & Methods [file 41598_2018_19845_MOESM1_ESM.pdf]

Starfish Apaf-1 activates effector caspase-3/9 upon apoptosis of aged eggs

Ritsuko Tamura[1], Mariko Takada[1], Miki Sakaue[1], Ayaka Yoshida[1], Shirabe Ohi[1], Kaoru Hirano[1], Tomoyo Hayakawa[1], Noritaka Hirohashi[1], Kei Yura[1], and Kazuyoshi Chiba[1,*]
[1]Department of Biology, Ochanomizu University, 2-1-1 Ohtsuka, Bunkyo-ku, Tokyo, 112-8610, Japan
[*]chiba.kazuyoshi@ocha.ac.jp

Supplemental Materials and Methods

Cytochrome *c* assay. Samples were incubated with 10 μM horse heart cytochrome *c* (Sigma) and 1 mM dATP (Invitrogen) final concentration for 60 min at 20°C. DEVDase activity was measured at indicated time by using Ac-DEVD-MCA.

Immunoprecipitation. Immunoprecipitation was performed according to the method described previously (Hiraoka *et al.,* 2004). Protein A Sepharose 4B (Sigma) was pre-adsorbed to the anti-caspase-3/9 antibody or rabbit IgG (Amersham). 20 μL of cell-free preparation was added to 2 μL of Sepharose beads, and incubated at 4°C for 120 min. The 2 μL pellet of Sepharose beads was used for western blotting.

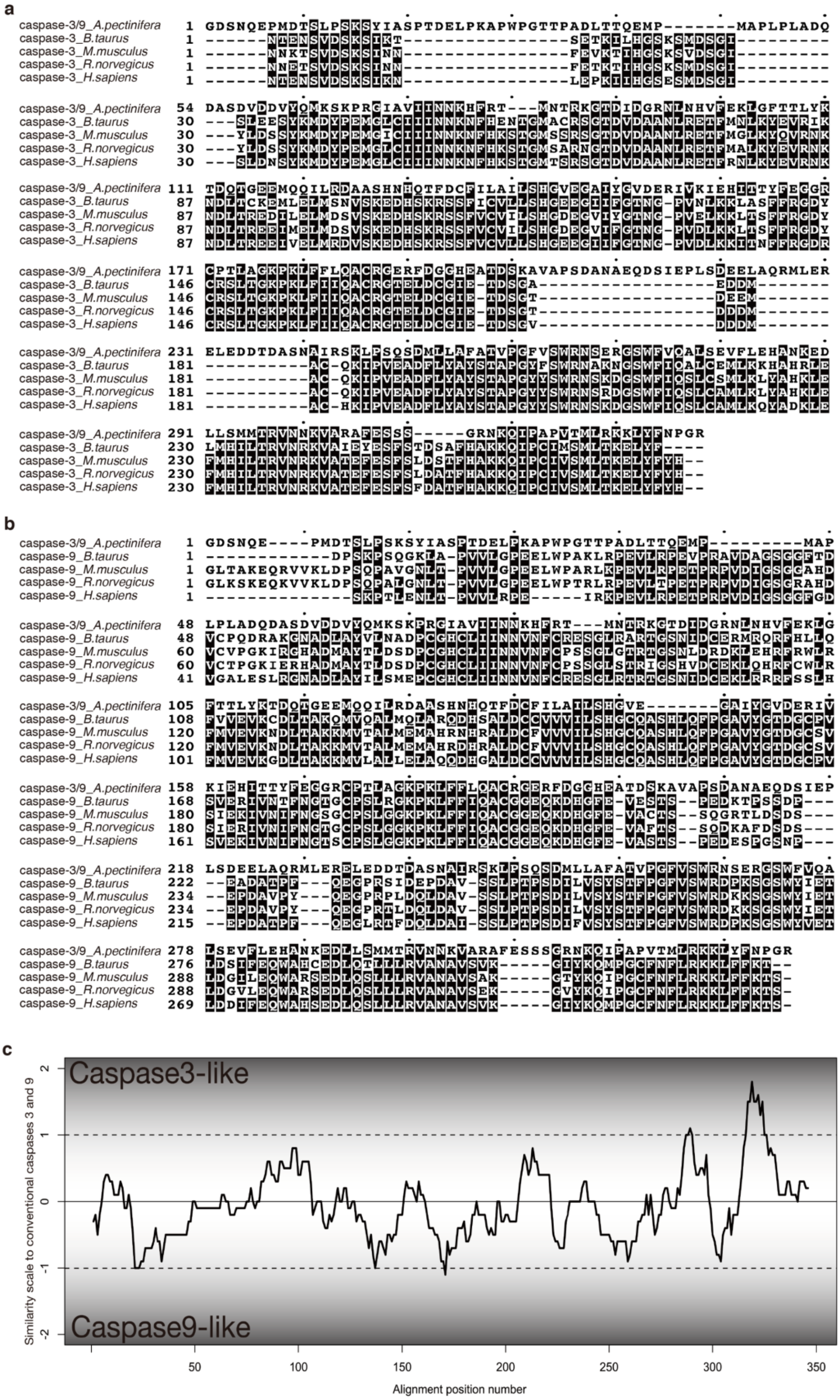

(the # of identical aa in caspase3) - (the # of identical aa in caspase9)

Supplemental Figure S1. (a, b) Multiple sequence alignment of caspase-3 (a) and caspase-9 (b) from *Homo sapiens*, *Rattus norvegicus*, *Mus musculus*, and *Bos taurus* against starfish, *Asterina pectinifera*, caspase-3/9. Positions with conserved amino acid residues are in a black background. The alignment was performed by MAFFT. (c) Conservation tendency graph of starfish caspase-3/9. The total number of black amino acid residues in a sliding window (every 15 amino acids) of caspase-3 (Supplemental Figure S1 a) was subtracted by the total number of black amino acid residues in the same sliding window of caspase-9 (Supplemental Figure S1 b). The subtracted number of each window was divided by 15, and obtained each value was plotted against the alignment position. A positive value means that the region in starfish caspase-3/9 has a caspase-3-like sequence and a negative value means that the region has a caspase-9-like sequence. The graph shows that starfish caspase-3/9 has a caspase-9-like sequence in the N-terminal side, and a caspase-3-like sequence in the C-terminal side.

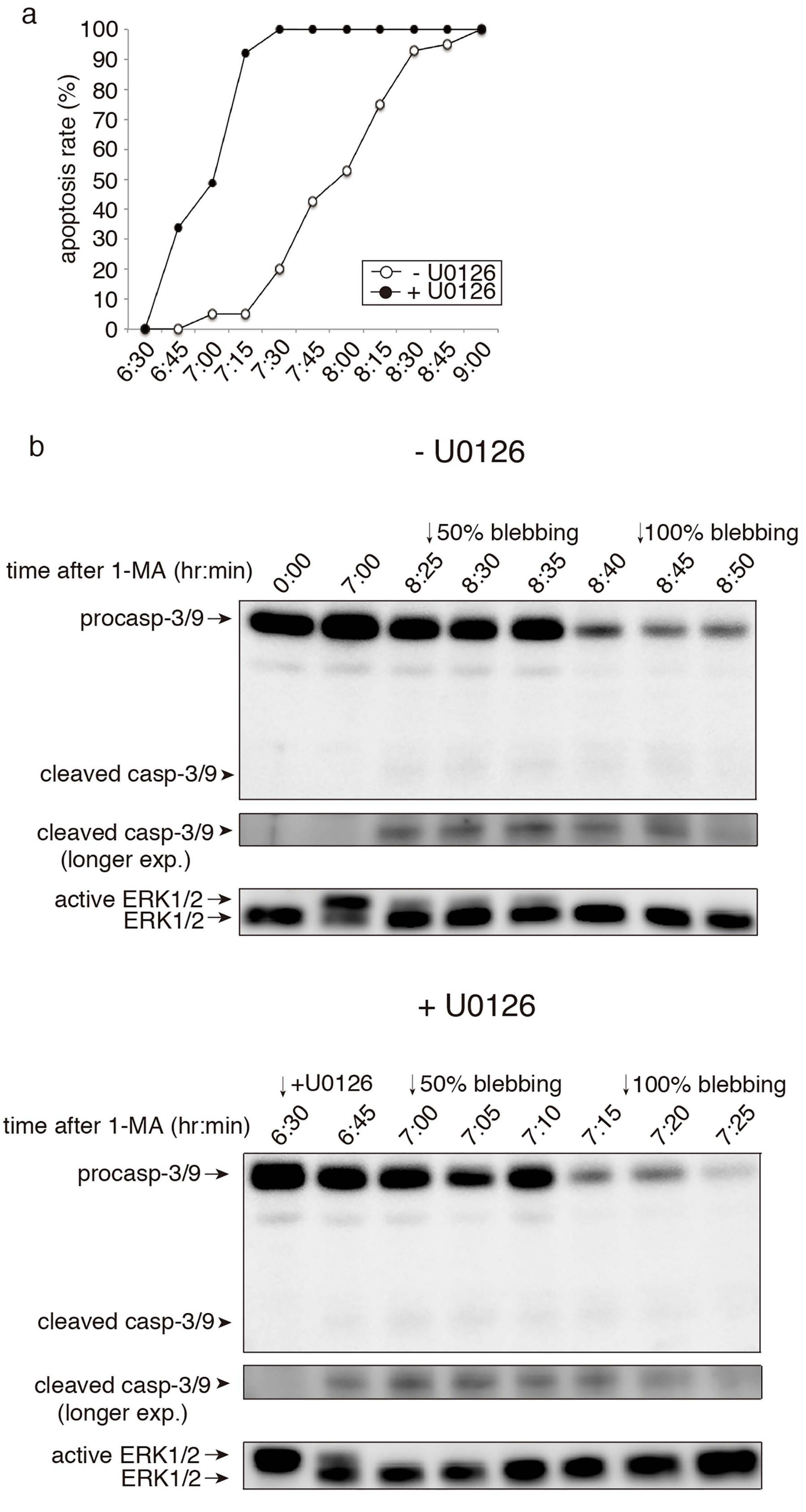

Supplemental Figure S2. Inhibition of ERK1/2 activates caspase-3/9. (a) The number of apoptotic eggs was counted at the indicated time after 1-MA treatment without U0126 (white circle) or with U0126 (black circle). Eggs were treated with 1 μM of U0126 at 6.5 h after 1-MA treatment. (b) Time course of endogenous caspase-3/9 activation with or without U0126. Samples were analyzed by SDS-PAGE and western blotting with anti-caspase-3/9 and anti-ERK1/2 antibodies. Cleaved caspase-3/9 was visible after longer exposures.

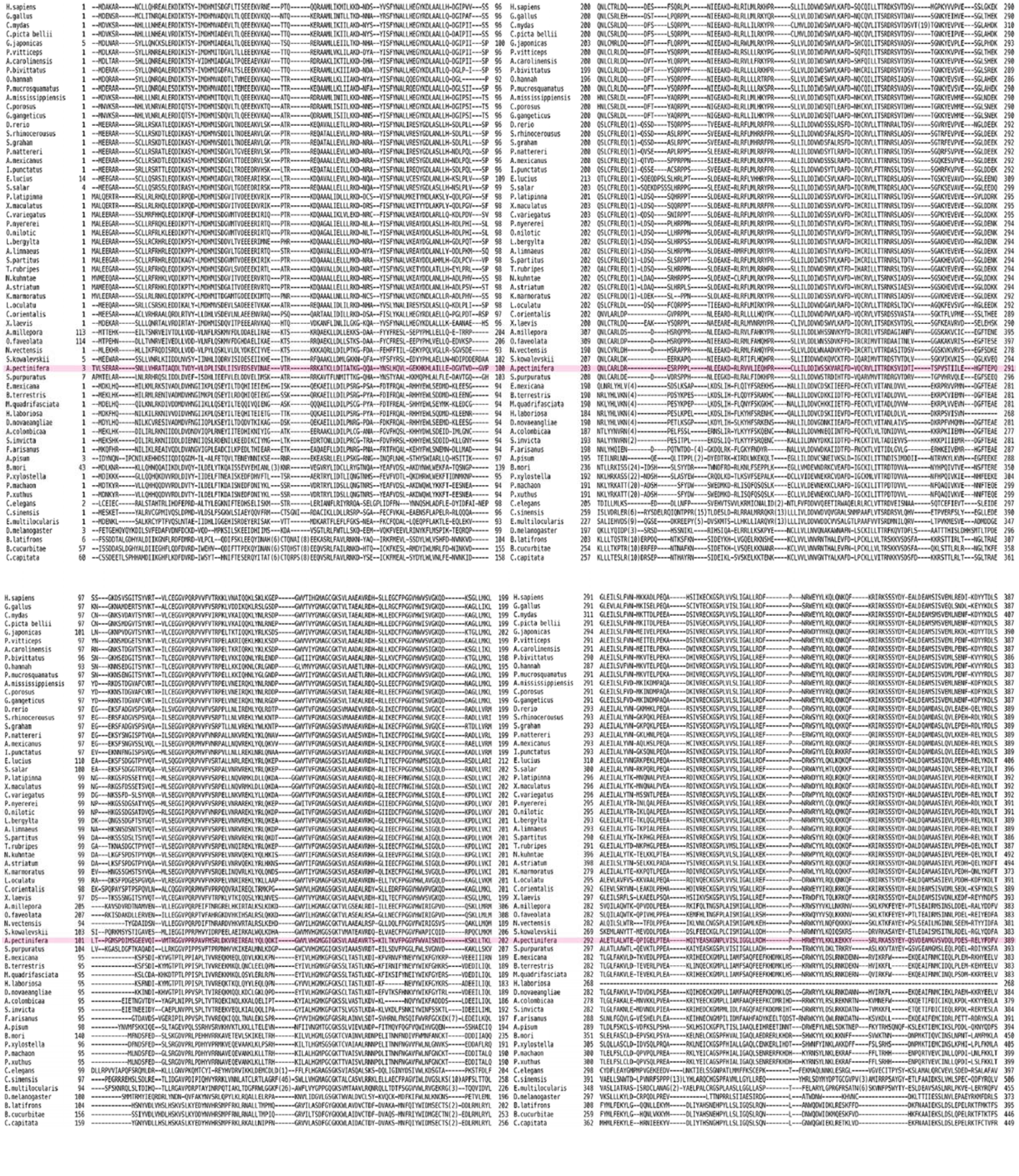

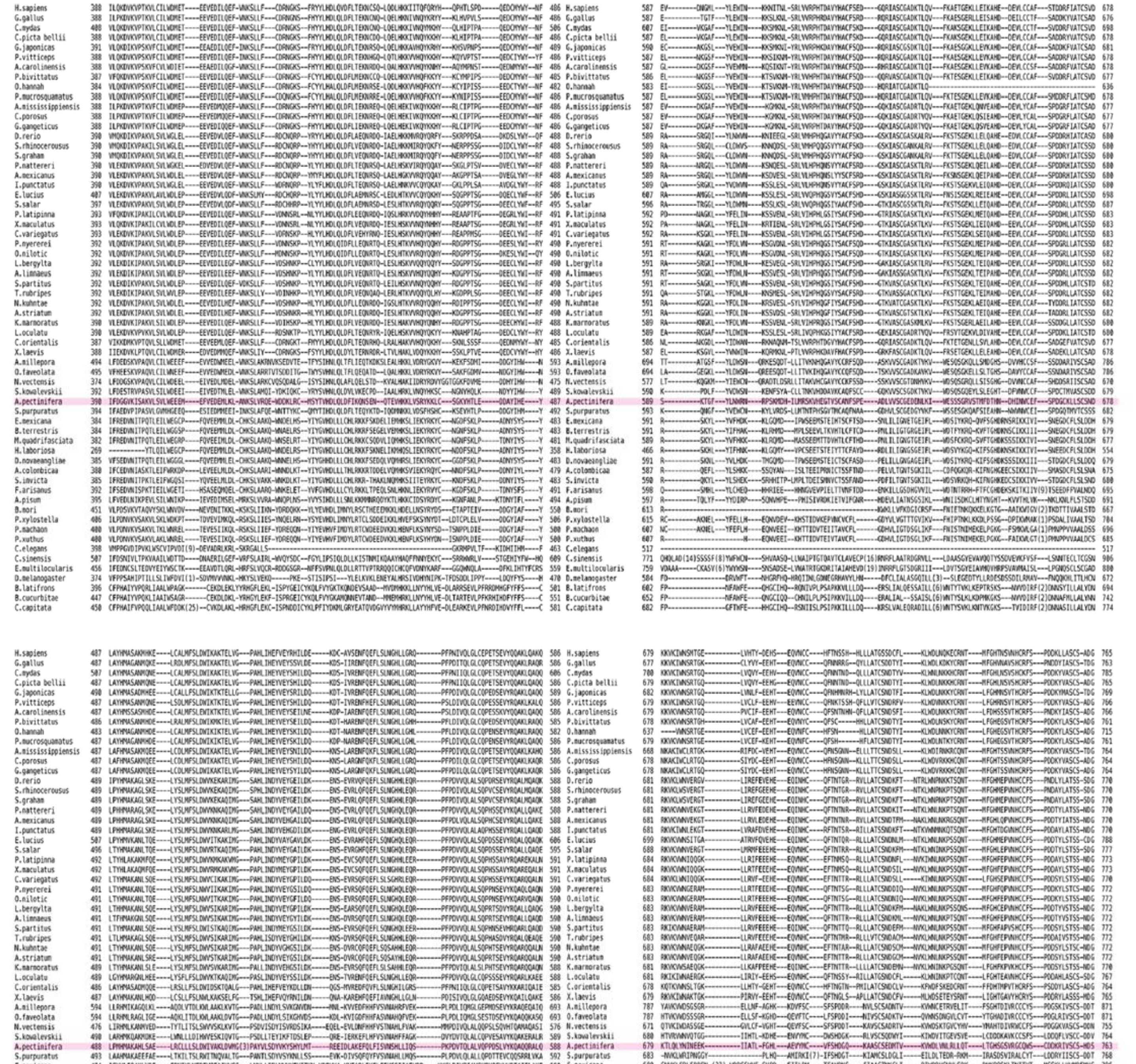

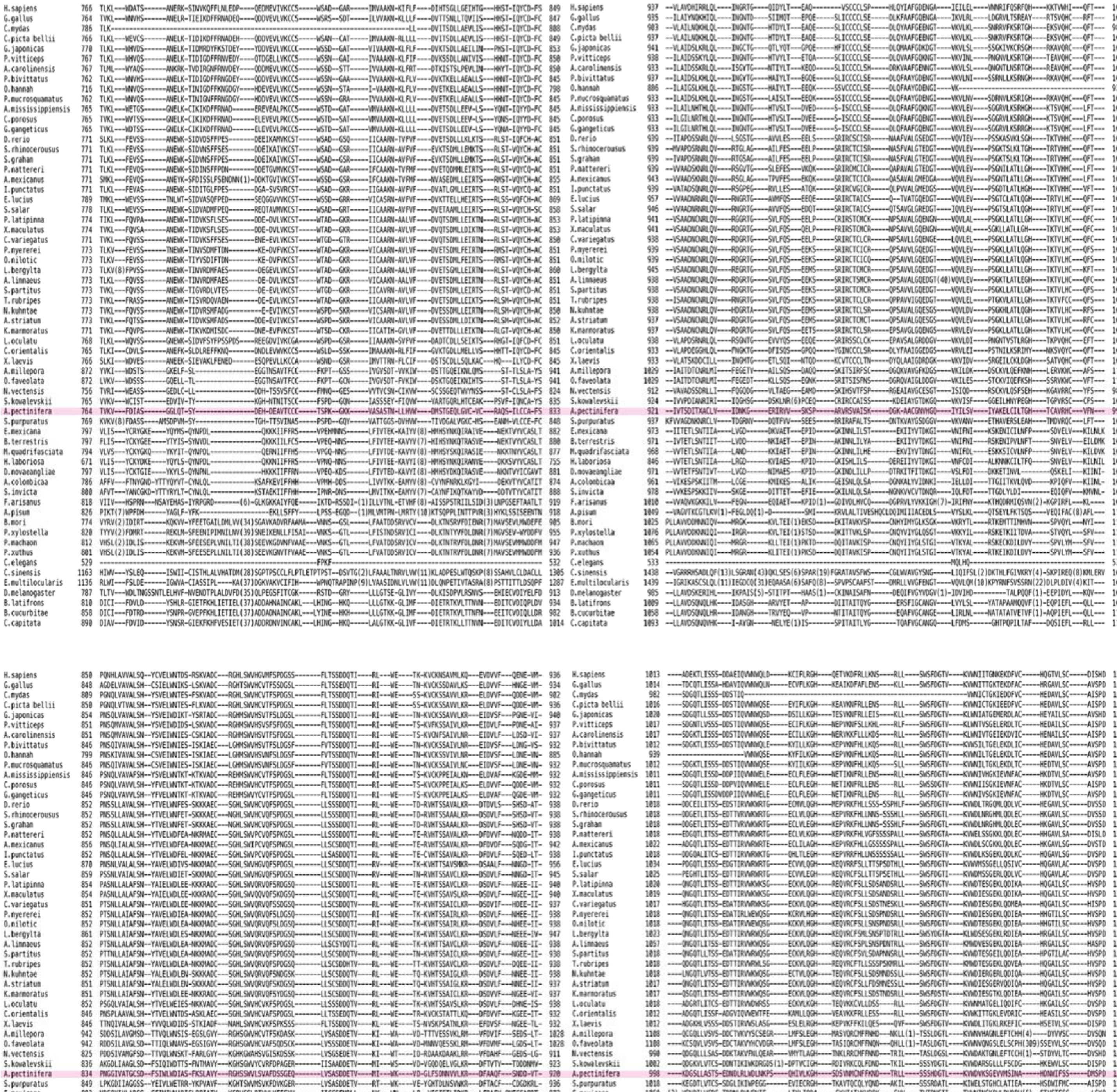

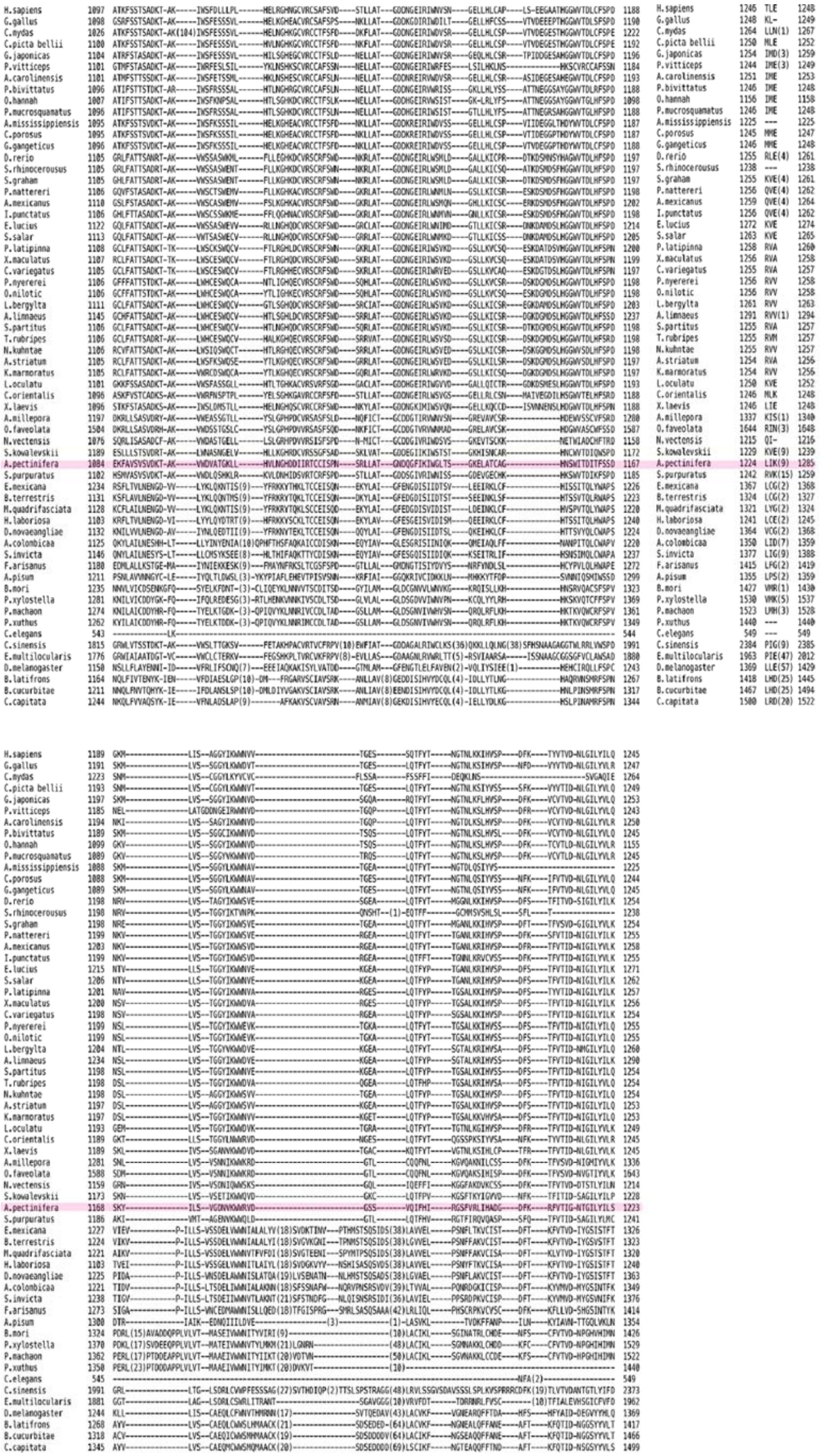

Supplemental Figure S3. Multiple sequence alignment of Apaf-1 family. The species used for alignment were mammals, birds, reptiles, fish, amphibians, cnidarians, hemichordates, echinoderms, insects, nematodes, platyhelminthes. Sequence alignment was generated using MAFFT, and the long insertions were deleted. The pink line on alignment indicates the sfApaf-1 sequence.

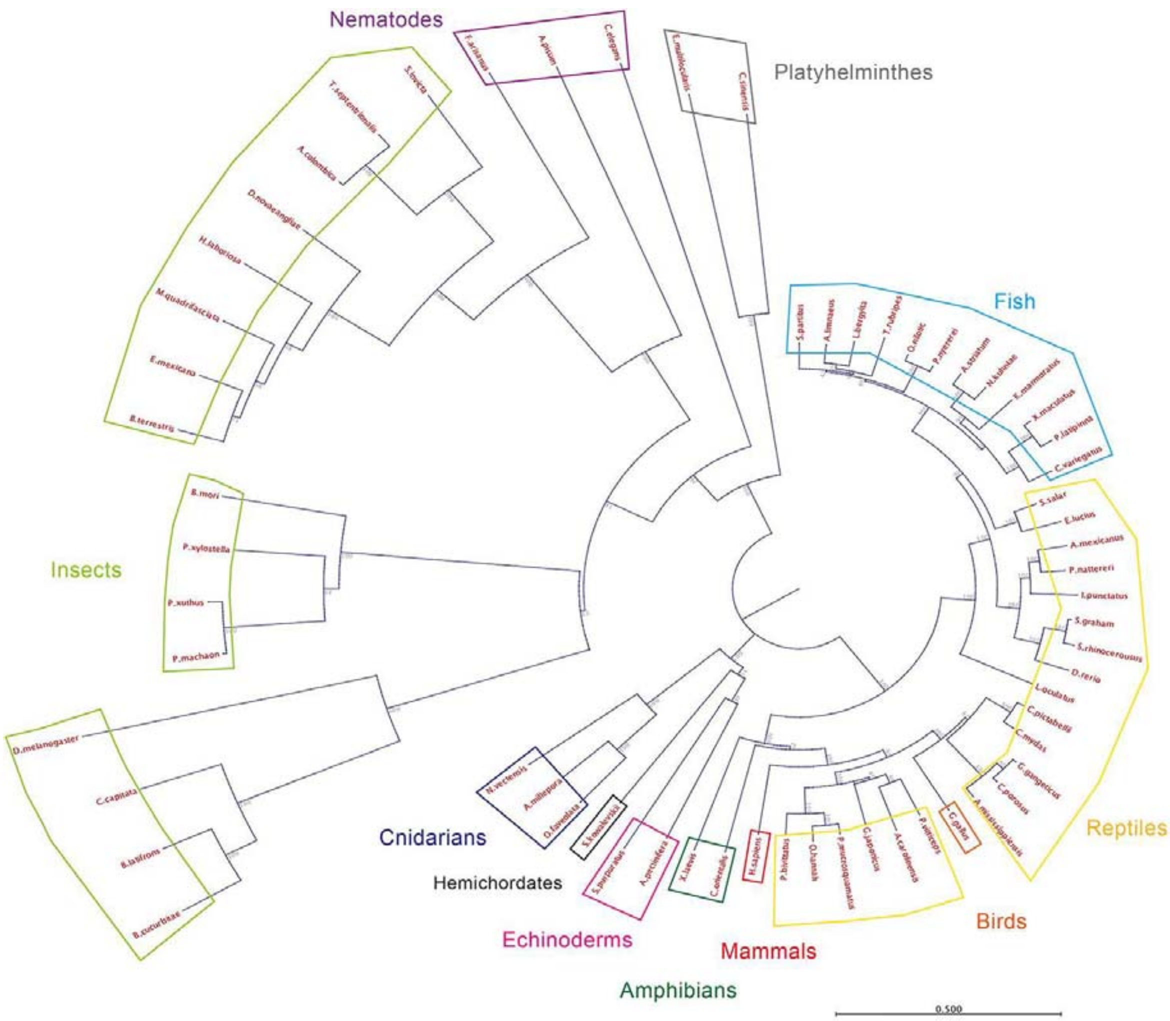

Supplemental Figure S4. Phylogenetic tree of Apaf-1 family proteins. The phylogenic tree was calculated using CLC Sequence Viewer ver.7.6 (CLC Bio A/S, Aarhus, Denmark) based on the alignment in Fig. S1. The species used for the alignment were mammals (red), birds (orange), reptiles (yellow), fishes (light blue), amphibians (green), cnidarians (blue), hemichordates (black), echinoderms (pink), insects (light green), nematode (purple), platyhelminthes (gray). A bootstrap value, a measure of the confidence in the branch, is attached to each node.

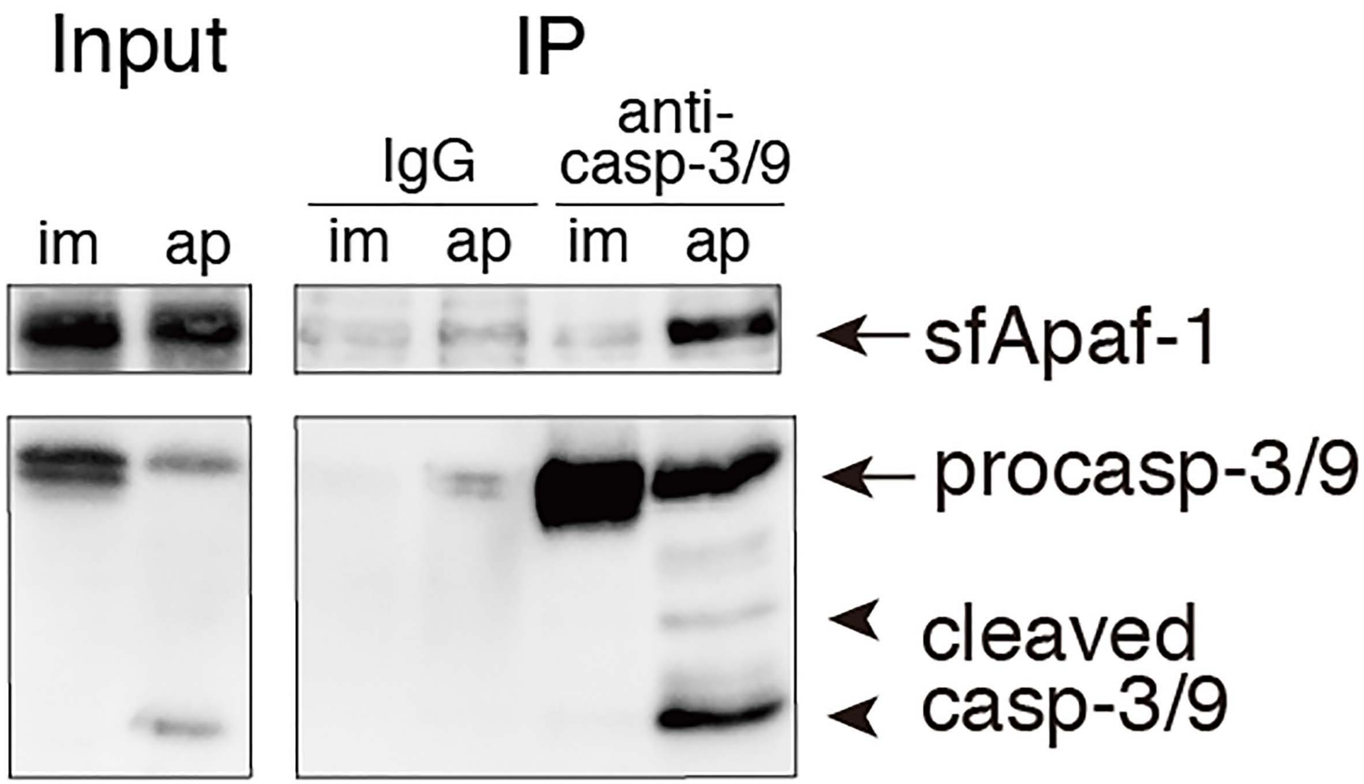

Supplemental Figure S5. Co-immunoprecipitation of endogenous sfApaf-1 and caspase-3/9. Cell-free preparations from immature oocytes (im) or apoptotic eggs (ap) were incubated with anti-caspase-3/9 antibody or control IgG-coupled Protein A Sepharose beads. Immunoprecipitates were analyzed by western blotting using anti-sfApaf-1 antibody (upper panel) or anti-caspase-3/9 antibody (lower panel).

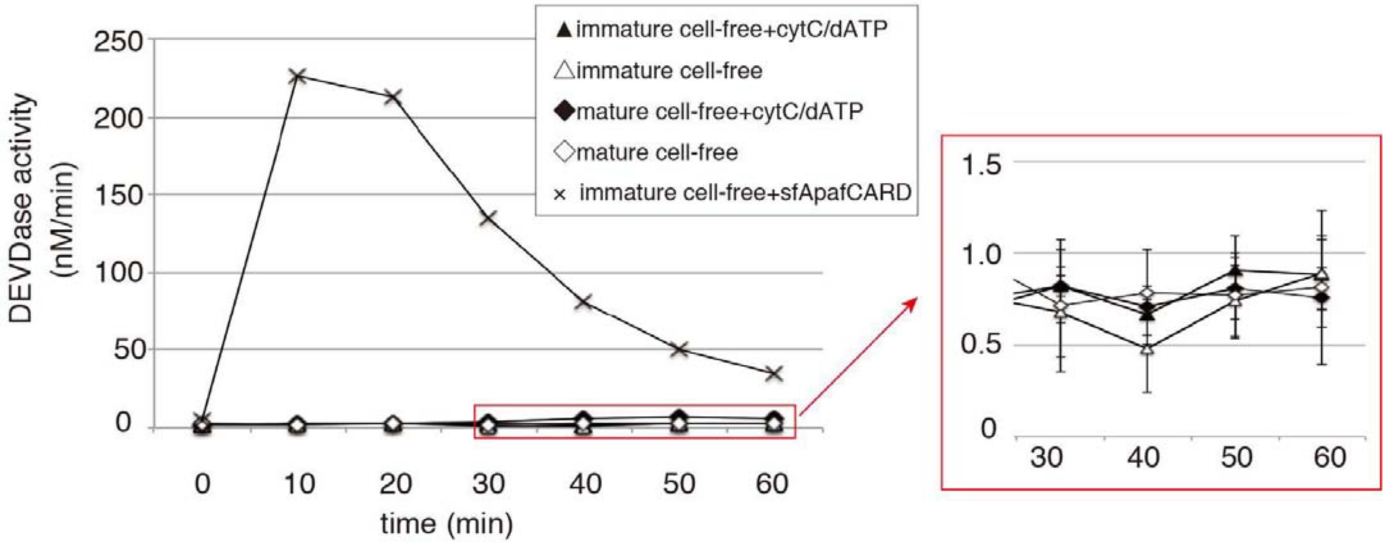

Supplemental Figure S6. Cytochrome *c* does not activate caspase-3/9 in starfish cell-free preparations. Cell-free preparations using immature oocytes and mature eggs were made according to the method of Chiba *et al.* (1999). Time course of DEVDase activity was measured in the presence (immature▲, mature◆) or absence (immature△, mature◇) of 10 µM cytochrome *c* and 1 mM dATP. Positive control experiments were performed in the presence of 2 mM GST-A-CARD (×).

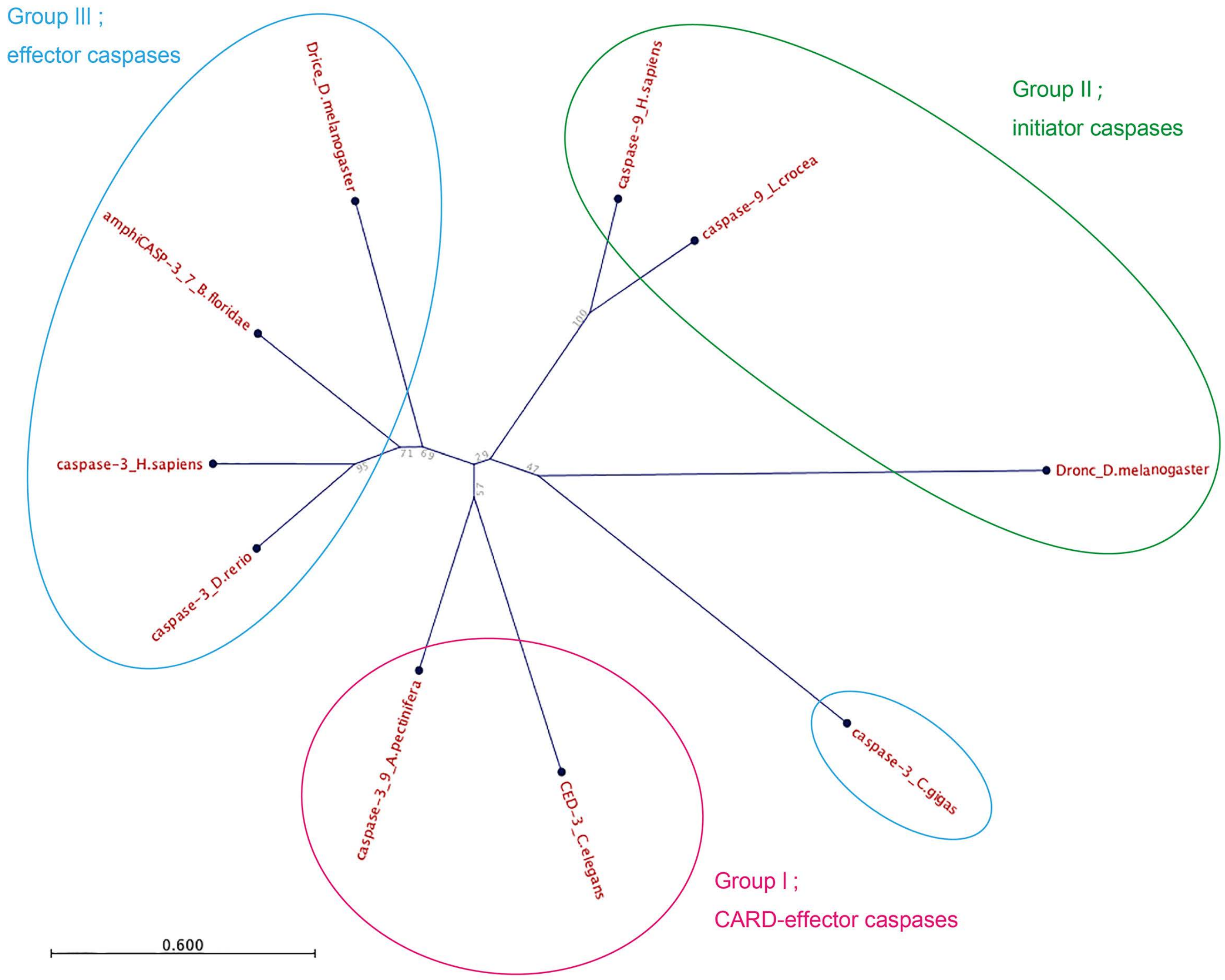

Supplemental Fig. S7. Phylogenetic tree of the caspases whose proteolytic activities were examined using recombinants. The phylogenic tree was calculated using CLC Sequence Viewer ver.7.6 (CLC Bio A/S, Aarhus, Denmark) based on the alignment which the long insertions were deleted. The species used for phylogenetic tree were *H. sapiens* caspase-3 (CAC88866) and caspase-9 (BAA87905), *D. rerio* caspase-3 (BAB32409), *Pseudosciaena crocea* caspase-9 (ACJ65026), *C.gigas* caspase-3 (XP_011449627), *B. floridae* AmphiCASP-3/7 (AAN45849), *C. elegans* CED-3 (AAG42045), *D. melanogaster* Drice (CAA72937) and Dronc (AAD26625), and *A. pectinifera* caspase-3/9. A bootstrap value, a measure of the confidence in the branch, is attached to each node.

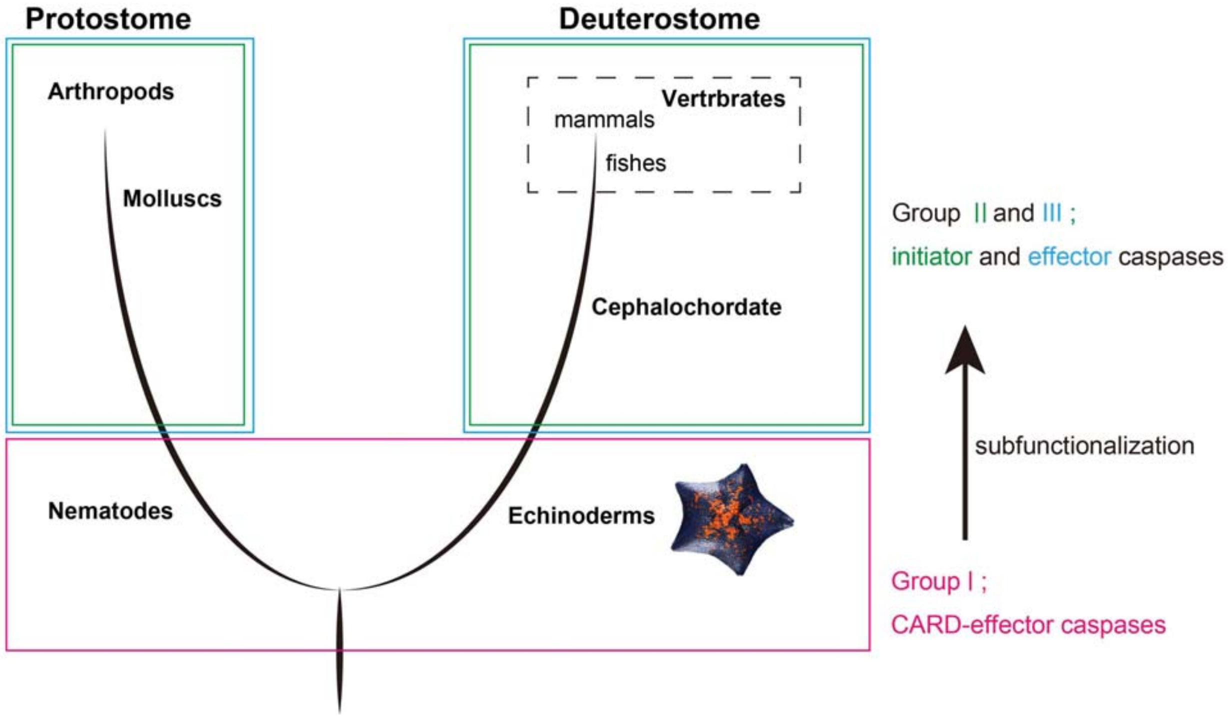

Supplementa Figure S8. Evolutionary trends of caspases. Conservation of caspases throughout animal evolution. Group I (CARD-effector caspases), Group II (initiator caspases), and Group III (effector caspases) are boxed in pink, green, and blue, respectively.

```
caspase-3/9      YIASPTDELPKAPWPGTTPADLTTQEMPMAPLPLADQDASDVD 178
caspase-9        VVLRPEIRKPEVLRP-ETPRPVDIGSGGFGDVGALESLRGNAD 150
                  :  *  . *:.  *  **  :   .  :. :   :.  .:.*
```

Supplemental Figure S9. Conservation of the MAPK consensus motif. MAPK consensus motif is boxed in starfish caspase-3/9 (136–178 aa) and human caspase-9 (109–150 aa). Sequence alignment was generated using MAFFT alignment program. Symbols at the bottom of protein sequences are indicated conserved sequences (*), conservative mutations (:), semi-conservative mutations (.), and non-conservative mutations ( ), respectively.

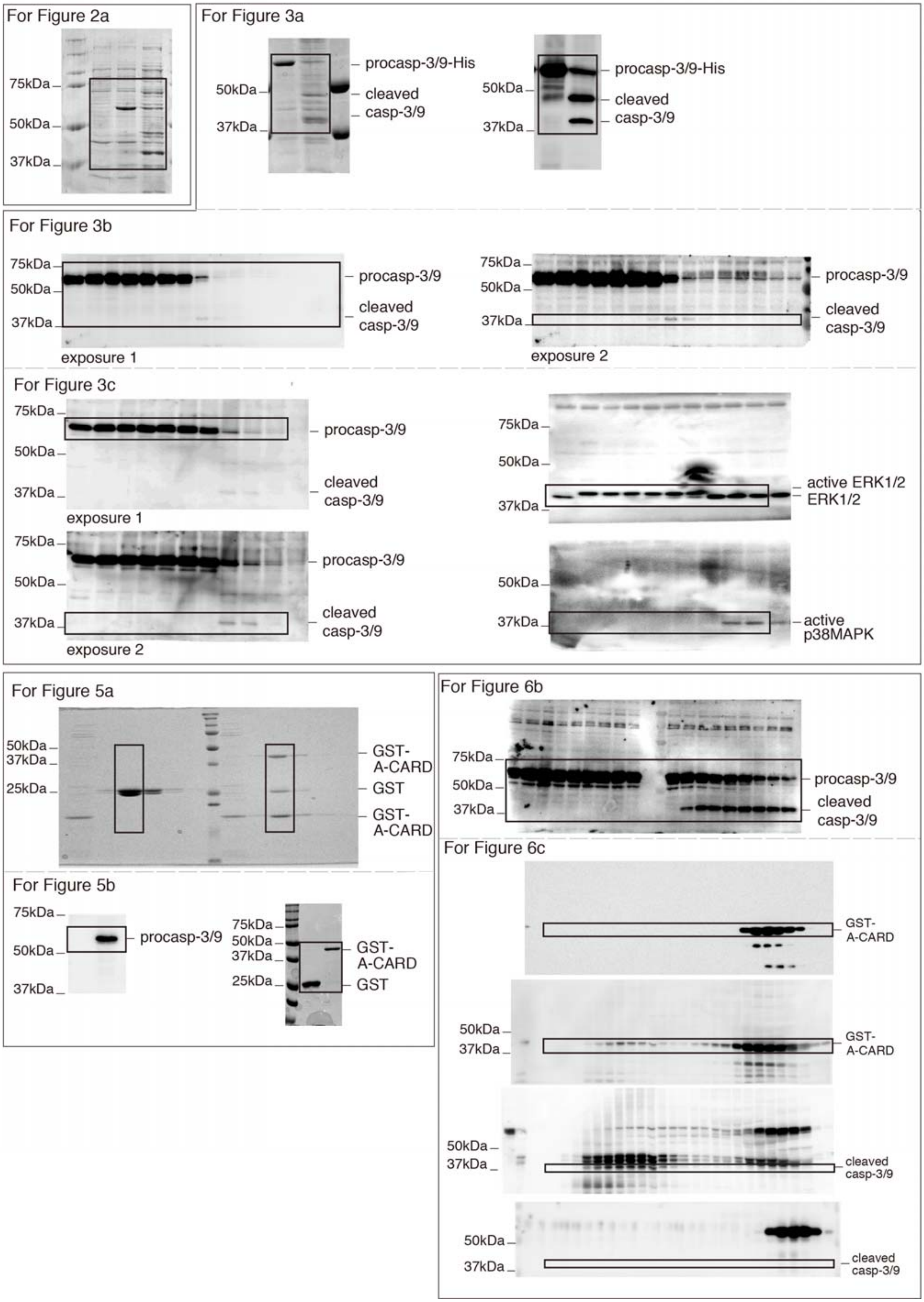

For Figure 2a
75kDa
50kDa
37kDa
For Figure 3a
50kDa
37kDa
procasp-3/9-His
cleaved
casp-3/9
50kDa
37kDa
procasp-3/9-His
cleaved
casp-3/9
For Figure 3b
75kDa
50kDa
37kDa
procasp-3/9
cleaved
casp-3/9
exposure 1
75kDa
50kDa
37kDa
procasp-3/9
cleaved
casp-3/9
exposure 2
For Figure 3c
75kDa
50kDa
37kDa
procasp-3/9
cleaved
casp-3/9
exposure 1
75kDa
50kDa
37kDa
procasp-3/9
cleaved
casp-3/9
exposure 2
75kDa
50kDa
37kDa
active ERK1/2
ERK1/2
50kDa
37kDa
active
p38MAPK
For Figure 5a
50kDa
37kDa
25kDa
GST-
A-CARD
GST
GST-
A-CARD
For Figure 5b
75kDa
50kDa
37kDa
procasp-3/9
75kDa
50kDa
37kDa
25kDa
GST-
A-CARD
GST
For Figure 6b
75kDa
50kDa
37kDa
procasp-3/9
cleaved
casp-3/9
For Figure 6c
GST-
A-CARD
50kDa
37kDa
GST-
A-CARD
50kDa
37kDa
cleaved
casp-3/9
50kDa
37kDa
cleaved
casp-3/9

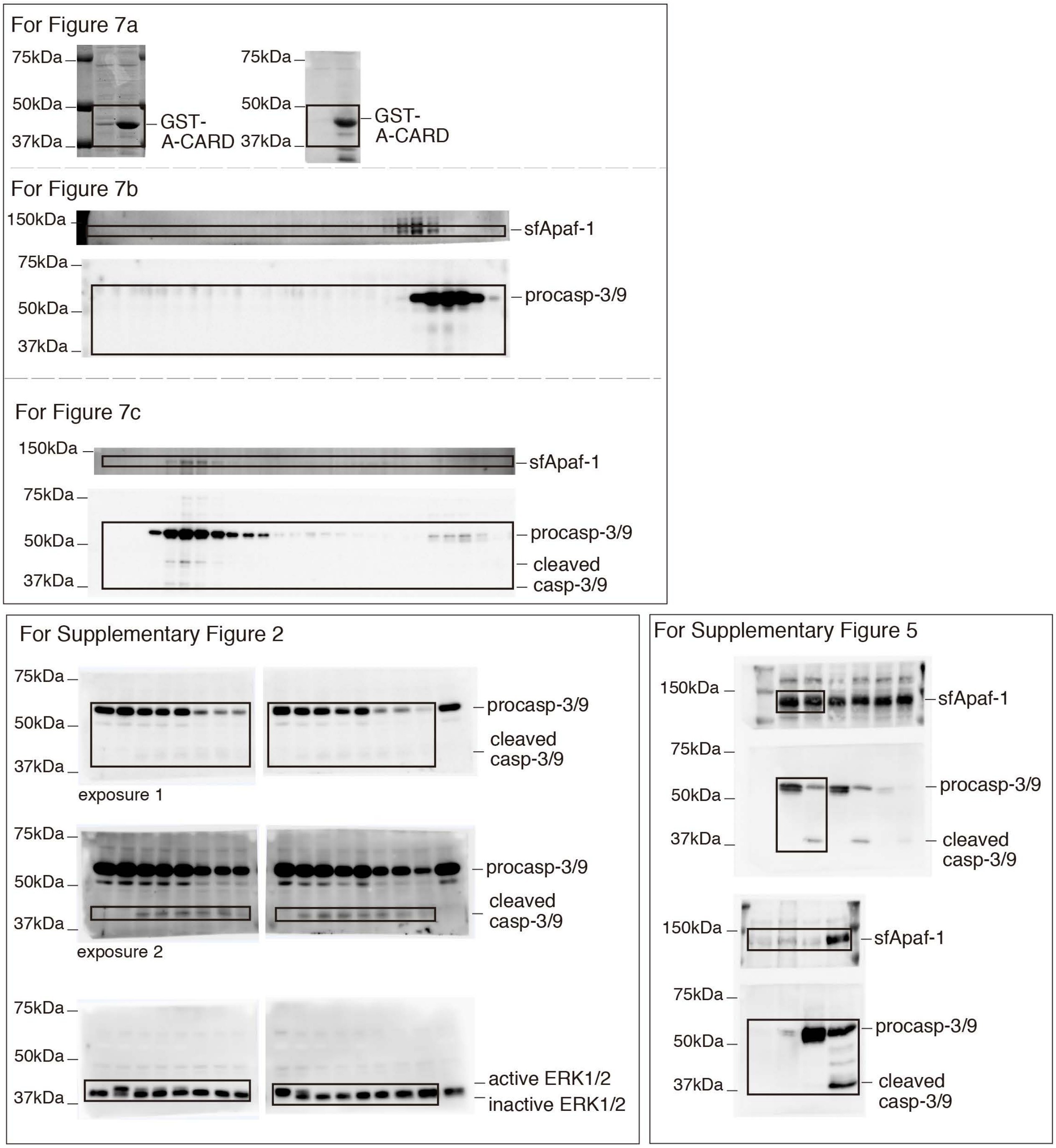

Supplemental Figure S10. Full-length gels and blots used for Figure 1-7 and Supplementary Figures.

Supplemental Table S1. Primers for cDNA cloning.

primer	sequences (5′-3′)	
degenerate primers for caspase-3/9	Fw	ATCATHAAYAAYAARAAYTTYSA
	Re	GCCTGRATRAARAANAGTTTRGGYTT
specific primers for caspase-3/9	Fw	TGGCAAGAACCTGAAGCACGTG
	Re	TCTGCTGCATTTGCTTCCCGGTC
degenerate primers for a partial sfApaf-1	Fw	GGNGGNGTNCCNATNCCICC
	Re	ARRAARTCNARYGNARRTYRTG
degenerate primers for a partial sfApaf-1 for nested PCR	Fw	CATGGNATGGGNGGNATNGGIAAR
	Re	ARRTARTAYTTCCANCKIKTIGG
specific primers for 5' RACE for cDNA transcription		[Phos]CTCAATCTATCCTT
specific primers for 5' RACE		CACAGGTTCTGTAGCTTGGT
		AGACTAGACCGTGAGTCC
specific primers for 5' RACE for nested PCR		CTGATGGCGACCCAGAACAC
		CCCACTCAACCTGGAGGAG
specific primers for 3' RACE		ATAAGCTGCGCTGCATGA
specific primers for 3' RACE for nested PCR		CAGGAATGGGATGATGATAAGCT
